# Integrating rapid risk mapping and mobile phone call record data for strategic malaria elimination planning

**DOI:** 10.1186/1475-2875-13-52

**Published:** 2014-02-10

**Authors:** Andrew J Tatem, Zhuojie Huang, Clothilde Narib, Udayan Kumar, Deepika Kandula, Deepa K Pindolia, David L Smith, Justin M Cohen, Bonita Graupe, Petrina Uusiku, Christopher Lourenço

**Affiliations:** 1 Department of Geography and Environment, University of Southampton, Southampton, UK; 2 Fogarty International Center, National Institutes of Health, Bethesda, MD, USA; 3 Department of Geography, University of Florida, Gainesville, FL, USA; 4 Emerging Pathogens Institute, University of Florida, Gainesville, FL, USA; 5 National Vector-borne Disease Control Programme, Windhoek, Namibia; 6 Department of Computer Science, University of Florida, Gainesville, FL, USA; 7 Clinton Health Access Initiative, Boston, MA, USA; 8 Department of Epidemiology, Johns Hopkins Bloomberg School of Public Health, Baltimore, MD, USA; 9 Mobile Telecommunications Limited, Windhoek, Namibia

**Keywords:** Human mobility, *Plasmodium falciparum* malaria, Malaria elimination, Migration, Disease mapping, Spatial analysis, Satellite imagery, Mobile phones

## Abstract

**Background:**

As successful malaria control programmes re-orientate towards elimination, the identification of transmission foci, targeting of attack measures to high-risk areas and management of importation risk become high priorities. When resources are limited and transmission is varying seasonally, approaches that can rapidly prioritize areas for surveillance and control can be valuable, and the most appropriate attack measure for a particular location is likely to differ depending on whether it exports or imports malaria infections.

**Methods/Results:**

Here, using the example of Namibia, a method for targeting of interventions using surveillance data, satellite imagery, and mobile phone call records to support elimination planning is described. One year of aggregated movement patterns for over a million people across Namibia are analyzed, and linked with case-based risk maps built on satellite imagery. By combining case-data and movement, the way human population movements connect transmission risk areas is demonstrated. Communities that were strongly connected by relatively higher levels of movement were then identified, and net export and import of travellers and infection risks by region were quantified. These maps can aid the design of targeted interventions to maximally reduce the number of cases exported to other regions while employing appropriate interventions to manage risk in places that import them.

**Conclusions:**

The approaches presented can be rapidly updated and used to identify where active surveillance for both local and imported cases should be increased, which regions would benefit from coordinating efforts, and how spatially progressive elimination plans can be designed. With improvements in surveillance systems linked to improved diagnosis of malaria, detailed satellite imagery being readily available and mobile phone usage data continually being collected by network providers, the potential exists to make operational use of such valuable, complimentary and contemporary datasets on an ongoing basis in infectious disease control and elimination.

## Background

Significant progress is being made in reducing the morbidity and mortality attributed to malaria globally [1-10], and the Global Malaria Action Plan (GMAP) [11] articulates a long-term vision for malaria eradication through shorter-term local efforts to eliminate malaria. A total of 36 of the 107 malaria-endemic countries have declared they have a national policy for malaria elimination or are pursuing spatially progressive elimination within their borders [11-14].

Achieving elimination requires a re-orientation away from the sorts of universal prevention and treatment measures that define a control programme towards targeted operations, such as identifying residual transmission foci, focusing vector control or parasite-based attack measures to high-risk areas, identifying and curing both asymptomatic and symptomatic infections, and managing importation risk [15]. Many of these operational requirements can be facilitated by accurate and timely creation of risk maps. Such maps can help elimination programmes understand the epidemiology of a disappearing disease, and may allow proactive deployment of vector control measures to high risk areas to prevent local transmission and onward spread to other receptive areas, or suggest areas where active case detection may be used to identify and treat remaining parasite reservoirs [16]. Parasite rate-based maps for malaria have now been constructed [17,18], but infection prevalence is a poor metric for measuring malaria at very low levels of endemicity (below 5% parasite prevalence) due to the large sample size surveys required for precise measurement in such contexts [19]. In very low transmission environments, diagnostically confirmed malaria incidence provides a more useful measure than prevalence, and elimination-focused programmes are building capacity to rapidly provide such information, including the place of residence of cases [20]. Such a surveillance system is a crucial component of an elimination strategy, but achieving and maintaining elimination will require finding and curing infections that may be asymptomatic or may never come into contact with reporting health facilities [15]. Such infections can be identified through intensive proactive surveillance, but the generation of case-based risk maps at high spatial resolution has the potential to remotely identify regions in which transmission is likely to be occurring more quickly and at substantially lower cost.

Risk maps are essential for knowing where to attack malaria, but they are insufficient for a strategic elimination plan. Attacking strategically requires deploying the right measures in the right places, and doing so in a way that gains are not lost due to movement of people and parasites. For example, an attempt to eliminate malaria in Haiti in the 1960s through mass drug administration combined with DDT-spraying failed because the highly mobile population continually reintroduced parasites into areas that had just been cleared [21]. Understanding human movement, which can provide connections between disparate high-risk areas, is critical to designing appropriate elimination strategies and avoiding resurgence in post-elimination settings [22,23]. However, data on human movement patterns in malaria-endemic regions have been difficult to obtain, and often restricted to local travel history surveys or census-derived migration data [22]. The rapid global proliferation of mobile phones has presented unprecedented opportunities for measuring and understanding human movement dynamics. The retrospective analysis of billions of call detail records (CDRs), whereby temporal sequences of phone tower locations through which user communications were routed are converted into movement trajectories [24-27], providing information on human travel for sample sizes of millions and at scales of entire countries. Previous studies have demonstrated the value of such data when combined with parasite prevalence maps in providing quantitative guidance to malaria programmes [25,28,29], and mapping ‘source’ and ‘sink’ areas of net infection export or import [30]. However, in elimination settings where infection prevalence is an inappropriate measure and where case-based malaria maps are of greater utility, such approaches have yet to be applied.

Here, the potential of integrating mobile phone CDRs with rapid case-based mapping in providing a dynamic evidence base to support malaria elimination planning in low transmission settings is demonstrated, using Namibia as an example. Between 2004 and 2011, scale up of vector control and case management interventions in Namibia contributed to a remarkable decline in reported malaria cases from 610,800 to 14,400 [31]. Namibia is rapidly scaling up its malaria programme, with significant strengthening of its diagnosis and surveillance systems planned over the next five years, focused on achieving elimination by 2020. While the country has a clear strategic plan and recently drafted national elimination policy in place [32], achieving its goals will require a clearly defined strategy to deploy resources to optimal effect. The integration of movement data with case-based risk maps for Namibia provides a dynamic framework for understanding the connectivity between existing and potential malaria risk areas and defining ‘source’ and ‘sink’ regions, where relatively larger numbers of parasites may be exported than imported through travel, and *vice-versa*. Targeting aggressive attack measures to source communities will reduce malaria both at their locations and throughout the wider region to which it exports parasites. At the same time, sustainable measures to reduce receptivity in sink regions will be important to limit onwards transmission from imported infections.

## Methods

### Ethical approval

This project was approved by Ethics and Research Governance of the University of Southampton (submission #7696).

### Mapping malaria risk

**Figure 1:**
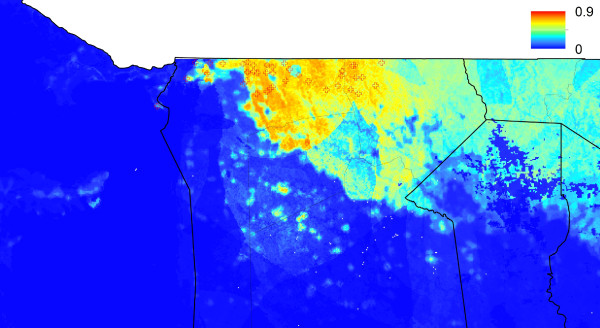
**Predicted probability of malaria cases in January-May 2011 for Omusati region.** The residential location of RDT confirmed cases are mapped as crosses.

**Figure 2:**
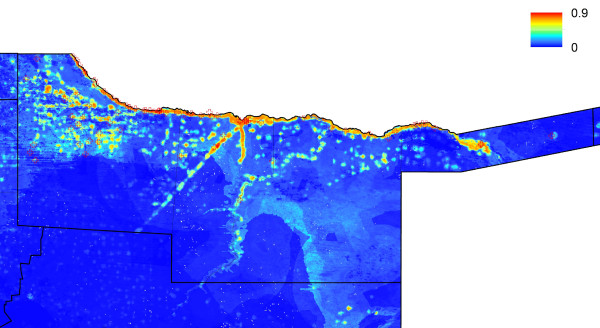
**Predicted probability of malaria cases in January-May 2011 for Kavango region.** The residential location of RDT confirmed cases are mapped as crosses.

**Figure 3:**
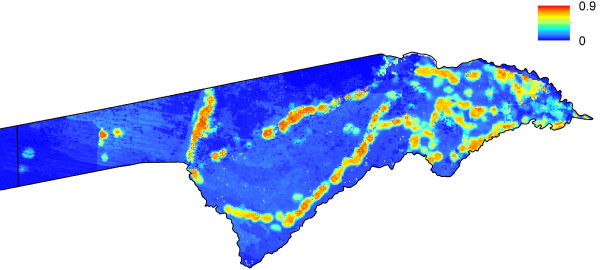
**Predicted probability of malaria cases in January-May 2011 for Caprivi region.** The residential location of RDT confirmed cases are mapped as crosses.

**Figure 4:**
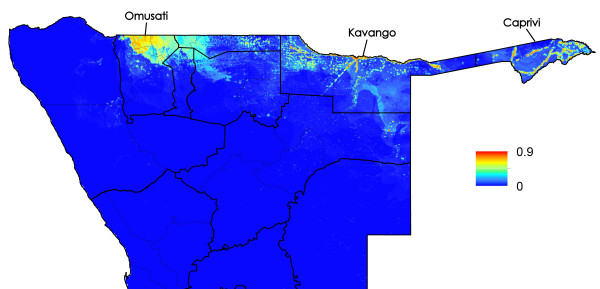
Predicted probability of malaria cases in January-May 2011 for northern Namibia (with named regions marked).

De-identified data on cases of malaria confirmed using rapid diagnostic tests (RDTs) reporting to health facilities across the three highest transmission regions, Kavango, Omusati and Caprivi (Figures 1, 2, 3 and 4) for the malaria transmission season in January to May 2011 were collected by the Namibia National Vector-borne Diseases Control Programme (NVDCP) in the course of routine surveillance. The community of residence of each patient, as reported to nurses at health facilities at the time of treatment, was geolocated. A total of 109 cases from 74 unique locations in Kavango, 234 cases from 41 unique locations in Omusati and 332 cases from 47 unique locations in Caprivi were successfully geolocated. The average age of cases across settlements and districts showed no systematic differences or biases. This indicated that transmission was likely not high enough in any location for significant immunity to develop and result in lower case loads due to immunity effects, rather than environmental drivers. The procedure for producing high resolution risk maps from the case location data followed closely that outlined in Cohen *et al*. [16] and is described below. Further details are provided in Additional file 1.

**Figure 5:**
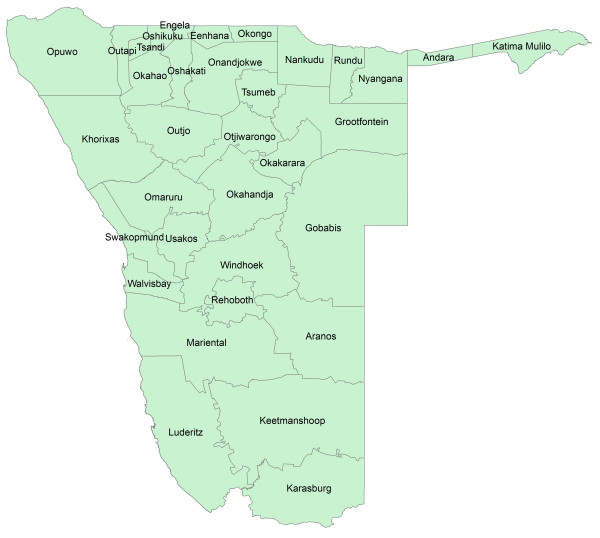
Health districts of Namibia.

**Figure 6:**
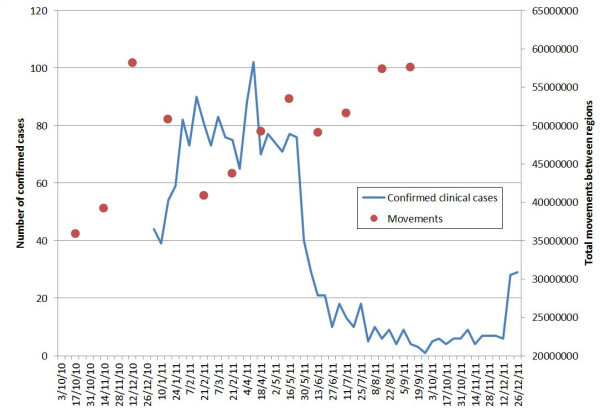
Plot showing the trends in total monthly phone-derived movements and reported malaria cases in Namibia.

Spatial covariate datasets representing rainfall, temperature, elevation, temperature suitability for *Plasmodium falciparum*, topographic wetness, vegetation, land cover, distance to water, infrastructure, and population density at 250 m resolution were assembled. Full descriptions and details of dataset sources are provided in Additional file 1. As transmission in Namibia is strongly seasonal, where covariate data were available by month, data for the January-May period were used to match peak transmission, following assessment of malaria seasonality in Namibia from aggregated NVDCP surveillance system data (Figures 5 and 6, Additional file 1). Values for each of the covariates were extracted for the point locations of communities with confirmed cases and ‘background’ points, randomly selected from across populated areas of the regions, identified using a population density dataset [33], with points sampled only from grid cells with population estimates of >0.1 persons. Background points do not necessarily indicate the absence of transmission, but instead characterize the environment of the country [34] in the places where people live. Travel history information from patients were not available, therefore to attempt to control for the fact that patients may have obtained infections away from their place of residence, locations with (i) just one case, then (ii) with one or two cases, were dropped, based on the assumption that multiple cases in a location are more likely to be representative of local transmission, and the output results compared to the mapping run using all case data to examine how sensitive outputs were to the exclusion of these isolated cases. Samples of 10,000 background points [34,35] were selected for each region.

Following Cohen *et al.*[16], the regression tree classification approach ‘Random Forest’ [36] was applied using the R [37] package ModelMap to model the risk of cases occurring in each 250×250 m grid cell, both separately within each of the three regions where the case data originated, and combined to undertake mapping across the whole of northern Namibia. Regression trees create a series of rules to partition the data into a set of groups that are as homogenous as possible with respect to the outcome [38]. For example, one such rule might differentiate the locations of case households from those of control households based on elevation below a certain threshold, while another rule might further divide the data based on levels of vegetation within specific bounds. In the Random Forest approach, the data are repeatedly split according to many different branching ‘trees’ of this type, and the final prediction is made by averaging across all of the individual trees [36]. To assess the accuracy of model predictions, 80% of the observed cases were selected at random for training the algorithm, with the other 20% used for testing, with this repeated 100 times. All of the predictor variables were included in the fitting step to produce a model predicting the probability of cases occurring at a particular location as a function of the combined covariates. Model quality was assessed by examining calibration plots [39], the area under the curve (AUC) on receiver operating characteristic (ROC) graphs and correlation statistics [40]. The fitted model was then applied in conjunction with the 250 m spatial resolution gridded datasets of the predictive variables to generate a map of predicted high season case risk across northern Namibia.

### Mobile phone call data records

CDRs covering the 12-month period October 2010 to September 2011 were provided by the leading mobile phone service provider in Namibia, Mobile Telecommunications Limited (MTC), who reported 1.5 million subscribers in 2011, and a 90% market share [41]. The data were obtained through written agreements between the network provider, the NVDCP, and the Clinton Health Access Initiative (CHAI). Following previous studies [24-27,30], anonymized records aggregated to the level of cell towers were provided to ensure that it was impossible to identify individuals.

For each CDR from a call or text message, the caller and receiver (identified using an anonymous ID), the receiving tower ID that the call was routed through, and the date of the call were recorded. Across the 12-month period, a total of 9 billion communications from 1.19 million unique SIM cards were identified in the dataset, representing 85% of the estimated 1.4 million adult (aged over 15 years old) population of Namibia [42]. Recent data on the typical ages of mobile phone owners in Namibia were obtained from the Universal Service Baseline Study of the Communications Regulatory Authority of Namibia [41] and showed that while the majority of users were between 20 and 30 years old, there was a broad spread across age groups (Additional file 2). Moreover, recent analyses suggest that such biases may have a limited effect on general estimates of human mobility [43].

Movements within urban areas were not considered here, given the principal focus of this study on examining regional movement patterns. Therefore, phone towers and movements falling within the boundaries of urban extents mapped using the Global Rural Urban Mapping Project Urban Extent (GRUMP-UE) dataset [44], were aggregated so that only movements between different urban areas or between rural and urban areas remained in the analysed dataset. This reduced the dataset of locations, or phone catchment areas, from 626 to 402. While rates of cross-border movements could not be ascertained from the data, due to the network providers only operating a national-level network, those crossing over the border into Namibia from neighbouring countries commonly switch to a local SIM-card (MTC, pers comm). This meant that the movements of such travellers and migrants were captured in the dataset, although the anonymized nature of the CDRs meant that they could not be identified, nor their movements analysed separately from Namibian residents. Daily locations were calculated for the subscribers using the location of calls and texts at one of the 402 phone catchment areas across the country, following methods outlined in other similar studies [24-27,30]. Subscribers were assigned a catchment area as their ‘home’ residence by where the majority of nights were spent throughout the full 12-month period. Movements between areas were calculated by examining the temporal sequences of calls or texts sent/received by subscribers and assigning a movement to a new area and a time of this move when the area through which their call/text was routed changed. Further, a general measure of population mobility, the ‘radius of gyration’ [24] was calculated for comparison of mobility differences between areas. The radius of gyration measures the characteristic distance travelled by a user over a certain time period (in this case, the 12-month period), and has been widely used in other CDR-based human mobility studies [24,26,45].

The mobile phone data processing outlined above enables construction of a weighted network of movements between each phone catchment area. The identification of distinct communities within this weighted network was undertaken using a modularity optimization algorithm [46]. The approach finds high modularity partitions of large networks and unfolds a complete hierarchical community structure for the network. In simpler terms, the approach identifies groups of areas that are connected by high levels of movement and combines them into a single ‘community’. Rates of movement within communities are generally higher than between separate communities. Such community detection approaches have been used in previous malaria studies to identify communities of regions that are either strongly connected by human or parasite movements, or are more isolated [47,48]. The community detection algorithm was run here on the networks of human and case risk scaled (see below) movements, and the differences examined.

### Population and malaria flows and connectivity

Movements of people and their infections were estimated for two types of travellers, following previous approaches [25,29,30]: (i) ‘Returning residents’: Residents of a location who visited a risk area then returned to their home location, potentially bringing an infection with them, and (ii) ‘Visitors’: Residents of a risk area who visited a new location and potentially carried an infection with them. Here, given the malaria case data available, the relative strengths of connectivity between locations in terms of the case-based malaria risks were examined, rather than attempting to estimate absolute numbers of infections moving.

For returning residents, it was assumed that the risk of acquiring an infection at their place of visit is a function of the level of risk at the visited location and the length of stay [25,29]. Therefore, a simple metric of cumulative risk was calculated by scaling the number of days spent at the visited location during the malaria transmission season months (January-May) by the modelled risk value there for each returning resident trip. For visitors to new locations during the transmission season, it was assumed that the relative risk of each visitor carrying an infection can be quantified by the estimated level of risk at their home locations. These simple metrics defined importation risk flow networks for returning residents, visitors and, by combining the two, overall risk flow, which quantified the connectivity through human movement scaled by predicted risk across northern Namibia. Throughout the focus is on flows and connectivity between locations for the January-May 2011 period.

### Mapping ‘sources’ and ‘sinks’

Through repeated introduction of malaria, human movement can make it appear that an area is sustaining transmission. Targeting the relatively larger exporter communities (‘sources’) of infections with aggressive attack measures is likely to have an impact on the numbers of infections both at that location and in surrounding areas that are net importers of infections (‘sinks’). At the same time, sink communities with substantial potential for transmission represent places where receptivity-lowering activities, such as vector control, may be important to manage the risk of imported malaria on an ongoing basis. This sort of strategic deployment of interventions is likely to increase the effect of limited resources. The estimation of relative malaria risk connectivity matrices described above enabled identification of the net exporters (sources) or importers (sinks) per location.

## Results

### Case-based malaria risk mapping

Univariate analyses demonstrated the utility of the majority of the spatial covariates in distinguishing case locations from ‘background’ conditions (Additional file 1). The Random Forest model provided further indication of this through strong model prediction performance with AUC = 0.96 and correlation = 0.82 (Additional file 1). Model assessments testing data and stratified by district confirmed the accuracy of the approach in its ability to identify locations of cases that were not included at the training stage (Additional file 1). Judging by the relative influence on the model predictions, outputs were most dependent upon the spatial covariates that quantified vegetation amounts, population density, precipitation, and presence of water. Least important variables were those related to temperature, elevation and remoteness. Results were similar when broken down by district (Additional file 1), highlighting the consistencies in likely environmental drivers of transmission across northern Namibia. Moreover, results appeared insensitive to dropping locations with only one or two cases (Additional file 1). Figures 1, 2, 3 and 4 depict the maps generated from the predictive model for the entire northern Namibia region, while Additional file 1 provides further descriptions and data from the modelling. Table 1 provides population weighted risk estimates per health district.

**Table 1:** Summary statistics for each health district

**Health district**	**Pop 2011**	**% phone users**	**Mean risk**	**Mean RoG**	**Mean trip length**	**Mean no. trips**	**Move comm**	**Risk comm**	**Pop in risk >50% and top 50 source**	**Mean effect index**
Andara	31,469	25	0.07	93.83	0.86	72.17	13	8	9779	0.00382
Aranos	27,669	29	0.00	107.37	0.93	13.57	5	8	0	0.00000
Eenhana	104,313	32	0.15	60.40	0.66	26.03	3	1	0	0.00416
Engela	127,931	38	0.34	45.23	0.30	30.86	3	1	0	0.00641
Gobabis	95,225	31	0.00	78.36	1.11	3.56	16	5	0	0.00010
Grootfontein	32,296	58	0.02	79.35	1.43	12.80	14	6	0	0.00215
Karasburg	19,946	58	0.00	163.64	2.55	2.63	5	8	0	0.00000
Katima Mulilo	80,460	48	0.16	78.50	0.71	7.08	4	8	4773	0.00407
Keetmanshoop	37,138	60	0.00	113.79	1.28	4.70	5	5	0	0.00000
Khorixas	17,839	59	0.00	84.48	2.35	4.39	14	4	0	0.00085
Luderitz	24,890	88	0.00	202.86	1.97	41.56	6	1	0	0.00000
Mariental	23,358	60	0.00	104.40	0.95	6.08	7	4	0	0.00000
Nankudu	38,601	32	0.11	71.04	0.56	21.77	4	8	12505	0.00389
Nyangana	23,109	31	0.11	57.50	0.27	50.91	13	8	8064	0.00515
Okahandja	70,058	36	0.00	67.63	0.49	4.86	7	5	0	0.00000
Okahao	35,674	41	0.03	48.86	0.33	73.64	14	4	21	0.00165
Okakarara	18,120	57	0.00	63.17	0.70	20.53	16	5	0	0.00045
Okongo	17,560	46	0.12	128.16	0.46	63.76	2	1	2628	0.00355
Omaruru	32,738	27	0.00	70.62	0.81	10.16	15	4	0	0.00000
Onandjokwe	148,412	36	0.04	52.33	0.43	18.25	8	3	0	0.00192
Opuwo	29,300	40	0.00	67.26	3.41	4.98	12	4	0	0.00155
Oshakati	152,355	76	0.07	51.55	0.40	17.86	9	5	0	0.00339
Oshikuku	120,363	39	0.42	51.40	0.28	28.99	10	7	26829	0.00840
Otjiwarongo	44,708	68	0.00	73.59	0.62	4.66	14	4	0	0.00012
Outapi	63,890	58	0.16	59.00	0.67	28.21	11	7	49703	0.00559
Outjo	17,772	59	0.00	68.16	0.63	10.14	14	4	0	0.00091
Rehoboth	71,282	30	0.00	66.42	0.94	10.15	7	4	0	0.00000
Rundu	67,743	65	0.13	83.98	0.45	14.46	13	8	72777	0.01091
Swakopmund	57,071	86	0.00	109.49	0.88	9.03	15	4	0	0.00000
Tsandi	33,510	20	0.24	45.31	0.32	121.86	11	7	6127	0.00364
Tsumeb	20,535	100	0.02	65.76	0.94	5.73	14	6	0	0.00582
Usakos	12,174	83	0.00	77.60	0.65	14.91	15	4	0	0.00000
Walvisbay	57,337	107	0.00	120.11	1.15	6.66	15	7	0	0.00000
Windhoek	357,909	90	0.00	82.04	0.76	1.50	7	5	0	0.00000

Health districts are mapped in Figure 5. The shortened column titles refer to the following: Pop 2011 = number of people estimated to be residing in each health district in 2011; % Phone users = % of Pop 2011 population that were estimated to be captured in the CDR dataset, based on numbers of unique anonymous user IDs; Mean risk = mean population weighted predicted malaria case risk on a 0-1 scale; Mean RoG = mean radius of gyration [24] of movements derived from the phone data (See Additional file 2 for more details); Mean trip length = mean length of trip taken in days away from home phone catchment area (See Additional file 2 for more details); Mean no. trips = Mean number of trips taken per year away from home phone catchment area (See Additional file 2 for more details); Move comm = movement community that the majority of the area of each health district belongs to (Additional file 2); Risk comm = malaria risk community that the majority of the area of each health district belongs to (Additional file 2); Pop in risk >50% and top 50 source = Number of people residing in areas where risk values are >0.5, and that are in the top 50 ‘source’ regions (Figure 9); Mean effect index = mean value of the effect index mapped in Figure 12.

### Human mobility

**Figure 7:**
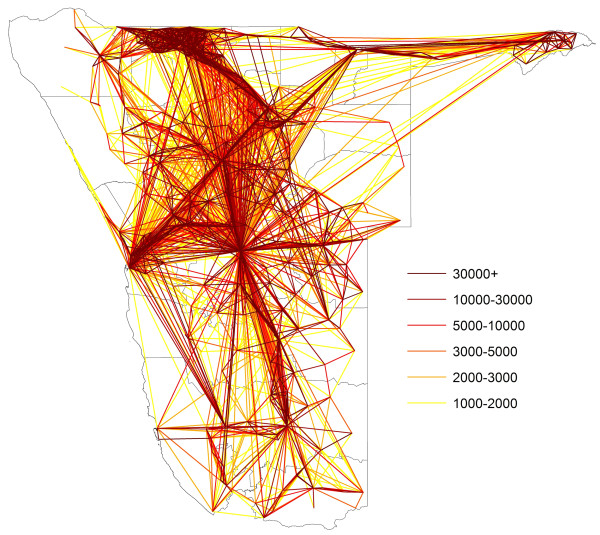
Movement totals between health districts over Oct 2010-Sept 2011 period, with rates of movement coloured from yellow (lowest) to red (highest).

Analyses of radius of gyration (Additional file 2) show that population movements in Namibia follow patterns seen elsewhere [24,26,45] of shorter distance movements being substantially more common than larger ones and more isolated populations generally travelling further than those in densely populated areas (Additional file 2). Across the 12-month period examined, a broad trend of increasing phone usage is evident (Figure 6), with some seasonality in overall movement rates evident, including increased activity in December, just before the main malaria transmission season (Figure 6). Spatially, movements follow the major transport routes, with the largest amounts of movement seen within the relatively highly populated north-central region (Figure 7). Table 1 presents summaries of mobility statistics by health district, with health districts mapped in Figure 5.

### Sources, sinks and communities of human and malaria infection movements

**Figure 8:**
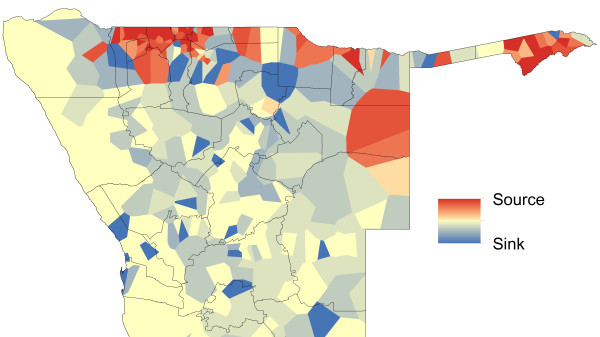
**Mapped ‘sources’ (net exporters) and ‘sinks’ (net importers) of malaria importation risk.** Areas coloured red are estimated to be net infection sources based on rates of movement and malaria risk, and those coloured blue are sinks, while those coloured yellow are neither substantial net exporter nor importers of infections.

**Figure 9:**
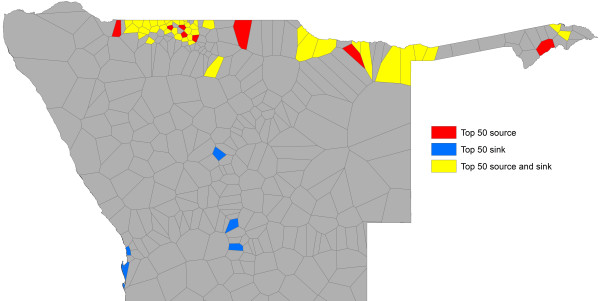
**Mapped ‘sources’ (net exporters) and ‘sinks’ (net importers) of malaria importation risk.** The locations of the top 50 sources and sinks by phone catchment areas.

Spatial heterogeneities in both movement patterns and predicted malaria risk translate clearly into variations in relative rates of infection movements, with phone catchment areas of strong net exportation (sources) located adjacent to areas that are net sinks (Figure 8). While there are differences between areas in terms of estimated net parasite importation and exportation, it is also clear that most of the northern region consists of areas that are simultaneously both major sources and sinks of parasites (Figure 9), as high movement rates drive parasite flows across the region. Unsurprisingly, the north-central border region, which has some of the highest predicted risks and largest, most mobile populations, also represents the largest source area for the country (Figure 9). However, with predicted malaria risk consistent across this region, heterogenities in movement patterns within it drive variation in risk connectivity, meaning that there are many regions, including most of the north, which are both net importers and have a high probability of cases seen (Figure 9). The substantial amounts of travel from Windhoek to the malarious northern regions and back, and from visitors to Windhoek from the north, make the capital the largest sink area (Figure 9). Community detection applied to the weighted networks of movements, and movements weighted by risk, between the 402 phone catchment areas resulted in differing sets of communities of strongly connected areas being found (Additional file 2), with spatial differences also apparent between returning residents and visitors (Additional file 2). Table 1 summarizes community membership by health district, with those districts within the same communities displaying stronger levels of internal connectivity through movements than between differing communities, providing guidance on which districts should prioritize coordinating surveillance and control efforts due to substantial population and parasite exchange.

### Spatial targeting

**Figure 10:**
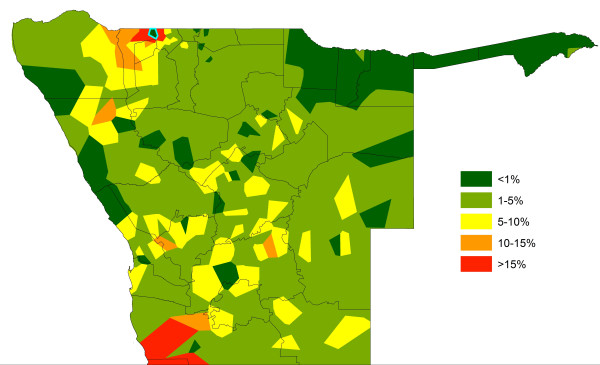
**The influence of connectivity through human mobility on the spatial impact of interventions.** The percentage reduction in importation risk through reducing parasite exportation numbers to zero in the phone catchment area marked in blue, which is one of the major source regions in Figures 8 and 9.

**Figure 11:**
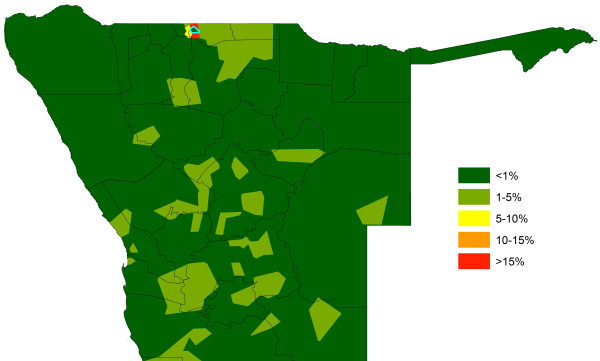
**The influence of connectivity through human mobility on the spatial impact of interventions.** The percentage reduction in importation risk through reducing parasite exportation numbers to zero in the phone catchment area marked in blue, which is of similar population size and risk level to the focus phone catchment area of Figure 10, but has lower movement rates.

**Figure 12:**
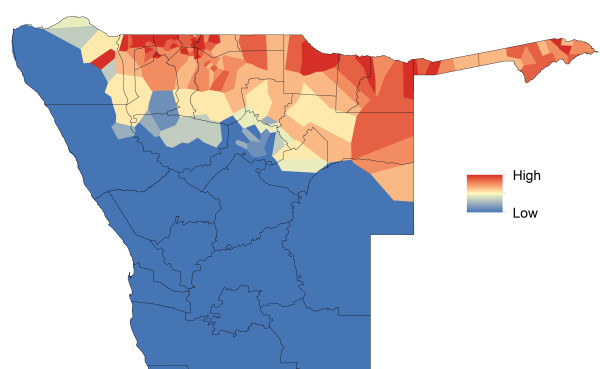
**The influence of connectivity through human mobility on the spatial impact of interventions.** Map of a ‘target effectiveness’ metric, which measures the relative reduction in importation risk elsewhere through controlling at each specific location, with red locations representing the areas where reducing parasite exportation to zero has the largest effects elsewhere, through to green, where minimal effects are seen. Health district names are shown in Figure 5.

The quantification of source/sink regions shown in Figure 3 enables theoretical scenarios on the impact targeting of control on malaria risk connectivity to be tested to guide attack strategies. Figures 10 and 11 demonstrate how differences in movement patterns can make a substantial difference in terms of regional impact on relative rates of case importation seen elsewhere through intervening in different areas. In Figure 10, a scenario is shown where the predicted malaria case risk at the phone catchment area highlighted is reduced to zero. As this is one of the largest source areas (Figure 9), the relative impact of this intervention is substantial across a wide region, with most impact within the malaria movement community it belongs to (Additional file 2). In contrast, the same intervention in a phone catchment area of similar population size and malaria risk, but lower mobility in terms of numbers and range of trips made to other catchment areas, shows a substantially smaller impact, both in magnitude and geographic extent terms (Figure 11). These intervention effects on relative impacts of infection exportation can be summarized through a simple ‘target effectiveness’ metric that measures, for each area, the relative levels of case importation elsewhere that would be stopped if malaria risk was reduced to zero in the area. This metric is mapped in Figure 12 and summarized by health district in Table 1, and shows a heterogenous pattern, indicating that the targeting of surveillance and control in certain areas may have a much larger impact on the surrounding region than in other neighbouring areas.

**Figure 13:**
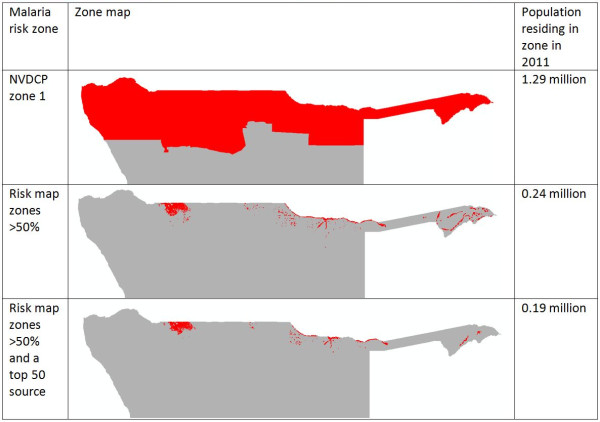
**Malaria risk zone maps and the size of populations to target according to the different categorizations.** The refinement of the mapped areas shows how the method can be used to target high-risk areas and populations, providing a method for prioritizing the delivery of limited resources.

Finally, Figure 13 demonstrates the utility of the combined mapping and movement quantification approach outlined here, through highlighting how high risk areas and populations could be prioritized for further investigation, surveillance and control. Existing national guidelines categorize the entire northern ‘zone 1’ region as the high-risk area where interventions should be focused. Through the rapid risk mapping approach, areas and populations within it can be highlighted that appear to be in particularly higher risk areas for cases. This refinement reduces the population to target from 1.29 million residing in the zone 1 region, to 0.24 million in the predicted higher risk zones. Within these zones, population movements mean that some areas are likely to be larger exporters (sources) of infections (Figures 8 and 9) than others, and the targeting of these can have a bigger effect on surrounding areas than the targeting of sinks (Figures 10, 11 and 12). Targeting only those populations residing in the major ‘source’ areas of the high risk zones, measured in this case by those phone catchment areas that are the top 50 largest sources (Figure 9), further reduces the focus population to 0.19 million.

## Discussion

As many countries reap the success of recent investments in malaria control with reported cases declining significantly, and re-orientate strategies towards elimination, parasite carriage by human travellers is rising up national and global agendas [14,22,48,49]. In elimination settings, the importation of malaria from outside a country becomes the focus of a malaria control programme, but intranational human population and malaria parasite movement is an important part of achieving elimination. Understanding this movement should be a critical component of the design of an elimination strategy, since it enables programmes to target resources in the most efficient way, plan attack strategies and ensure that context-adapted intervention strategies are employed across all high-risk areas. Past difficulties in quantifying and gaining a better understanding of human movement patterns are being overcome through new technologies [24,50] and here the potential of one of these, mobile phones, is outlined in providing valuable information that can be integrated with rapid case-based malaria risk mapping [16] to guide the design of disease control and elimination strategies.

The analyses presented here illustrate heterogeneities that exist in terms of both malaria risk and mobility across Namibia. The case-based risk mapping results (Figures 1, 2, 3 and 4) reveal the consistency in driving factors of the probability of cases at the spatial scales examined here between the three regions for which data were available (Additional file 1), as well as the accuracy with which risk factors and areas can be distinguished from the lower risk ‘background’ conditions (Additional file 1). Through integrating such high resolution risk mapping with CDRs, the targeting of elimination activities through identifying aspects of risk analogous to both the ‘hotspots’ and ‘hotpops’ concepts [49] could be undertaken if system flexibility and costs of undertaking this allow, enabling the focused deployment of limited resources in an attempt to focus surveillance activities and maximize impact (Figure 13). In planning an attack strategy, thinking spatially and accounting for mobility could be critical – with a mass drug administration (MDA) or mass screen and treat (MSAT) approach, reducing receptivity in high transmission risk sinks could be a focus through encouraging bed net use, while high transmission sources are attacked (Figures 1, 2, 3, 4, 8, 9, 10, 11 and 12). Such an approach will likely be much less costly and operationally difficult than trying to achieve blanket high coverage of MDA/MSAT in all high-risk areas (Figure 13). In post-elimination settings, the framework presented here provides guidance for targeting surveillance by highlighting how areas that are climatically, ecologically and demographically receptive to transmission are connected by human movement (Figures 8 and 9, Additional file 2) and through examining likely sources and onward movements from local outbreaks. It is clear that the exportation of parasites to other locations is not always problematic if the destination is not receptive, and the approaches presented here enable the separation of these ‘dead-end’ movements from possible problematic movements to receptive areas. The design of strategic plans for controlling, eliminating and preventing malaria re-establishment should, therefore, ideally account for human and, in turn, likely parasite movement patterns, and the analyses presented here show that tools built on the integration of datasets that are readily collected and stored by control programmes, satellite operators and mobile phone network providers can provide this valuable information for prioritizing efforts.

Whilst the analyses presented of the connectivity between risk areas in a malaria elimination setting go beyond previous work, it is clear that a range of uncertainties remain. Many of those crossing the border into Namibia will be captured by phone data due to SIM card switching, but clearly one of the biggest drawbacks of such data for mobility analyses is the lack of cross-border movement rate quantification. Infection importations from Angola and other neighbouring countries likely play a role in the epidemiology of malaria in Namibia [48], and if the community detection analyses could include cross-border movements they would likely highlight the north-central regions as being in the same community as south-central Angola and Caprivi joined with its surrounding countries, with many economic and family ties across the border prompting significant movements [51] and collaboration in control being vital if elimination is to be achieved [14,48]. While phone ownership and usage is high in Namibia, only a certain percentage of the population is being represented by the CDRs used here, and these are partially biased towards specific age groups and the richer and more mobile segments of the country [30,41] (Additional file 2). Moreover, the demographics and daily activities of network subscribers remain relatively unknown (Additional file 2), with different groups and activities likely presenting significantly greater risks of infection acquisition than others [22,47,52]. However, recent analyses on similar data in Kenya suggest that this is not likely to present a substantial bias in mobility estimates [43].

In terms of the risk mapping undertaken, it remains clear that the approach identifies broad areas of suitability for finding cases based on ecological, climatic, physical and demographic indicators, which provides no guarantee of finding ongoing transmission. However, the cross-validation undertaken suggests good performance in terms of identifying areas where cases have occurred (Additional file 1), providing a valuable tool for prioritizing areas for surveillance and further investigation. Ideally, alternative metrics of transmission, such as serological markers [53] should also be incorporated as more stable measures of transmission and to identify asymptomatic infections, thus, better quantifying true hotspots of transmission, but such measures are not yet routinely collected. The utilization of training data from just three districts here, where also spatial differences in treatment seeking rates remain unknown, results in uncertainties in risk predictions elsewhere, though the accuracies in predictions and consistency in variables selected as top predictors across the three districts suggests that the drivers of transmission remain relatively consistent countrywide (Additional file 1). Moreover, broad similarities of the outputs to the most recent surveillance data [31] also suggests accurate mapping prospectively. Assessment of the sensitivity of outputs presented here to variability in quality of surveillance system data should represent an area of future work, however. Ideally, information on the receptivity (the propensity to result in onward transmission following an imported case) of each area should form a valuable additional metric to improve assessments of local transmission risks from case introductions. Pre-control era prevalence data have been used to define this for the 1969-92 period for Namibia [54,55], but significant development, population growth and urbanization over recent years [42,56] have likely changed receptivity substantially. Finally, the lack of travel histories in the case data used raises the possibility that some infections were acquired away from their location of residence, though the strong clustering of cases is indicative of local transmission and removal of isolated cases left outputs unchanged (Additional file 1).

The continued upgrade of the Namibia surveillance system, as well as those in other elimination countries, will begin to provide more in-depth information on cases, enabling the separation of likely local *versus* imported cases, as well as the travel histories of imported cases [57]. These improvements in type, quality and quantity of surveillance data will in turn present opportunities for the application of improved space-time statistical mapping approaches and mathematical transmission models to quantify and account for uncertainties, as well as the estimation of post-elimination risks of resurgence [23]. As data become more regularly reported, a central repository in the form of an online mapping tool is likely to be an important asset for elimination programs [58,59]. Integrating into such a tool rapid case-based risk mapping that can be dynamically updated as new data are reported, to account for seasonal and interannual variations [16], would provide useful prioritization for further investigations and surveillance activities. The linkage to phone data would then provide valuable information on mobility and connectivity. Further, combining the CDRs with other forms of movement data, such as census, survey and satellite [22,50,60], could inform on the demographics, drivers and seasonality of movements, as well as cross-border data, all of which are lacking in phone data. Finally, many of the methods outlined here are not restricted to malaria elimination scenarios, with issues such as artemisinin resistance spread [61,62], vaccine-preventable childhood illnesses [63], and the elimination of other diseases [64] also reliant on an understanding of movement dynamics.

## Competing interests

The authors declare that they have no competing interests.

## Authors’ contributions

AJT, CL, JC, ZH, DKP, JC and DK designed the study. CN, PU, CL and AJT undertook malaria data collection and processing. GB, ZH, UK and DKP processed the mobile phone and household survey data. AJT, ZH and UK undertook the malaria risk mapping and phone data integration. All authors contributed to the writing of the manuscript and have read and approved the final version.

## Supplementary Material

Additional file 1 **Case-based malaria risk mapping – additional details.** Additional information on the datasets, methods and results for the case-based risk mapping. Click here for file

Additional file 2 **Mobile phone call detail records – additional details.** Further information on phone ownership, mobile phone network geography and mobility patterns in Namibia. Click here for file
