# Supplementary material for: Integrating rapid risk mapping and mobile phone call record data for strategic malaria elimination planning
Source: Malar J. 2014 Feb 10;13:52. doi: 10.1186/1475-2875-13-52 (PMC3927223; doi:10.1186/1475-2875-13-52)
Supplement: Additional file 1 — Case-based malaria risk mapping – additional details. Additional information on the datasets, methods and results for the case-based risk mapping. [file 1475-2875-13-52-S1.docx]

**Additional file 1: Case-based malaria risk mapping – additional details**

***Spatial covariates***

Following Cohen et al [[1](#_ENREF_1)], a range of spatial covariate datasets were assembled, and each is described below and documented in table 1, with images of selected datasets shown in figure 1.

Rainfall and temperature strongly impact the biology of malaria transmission [[2](#_ENREF_2)], while elevation and topography have been demonstrated to influence risk through their effects on temperature and suitability for mosquito breeding [[3](#_ENREF_3)]. Gridded mean, minimum and maximum rainfall and temperature data were obtained from the WorldClim project [[4](#_ENREF_4)]. A temperature suitability index (TSI) for *P. falciparum* transmission dataset was also obtained [[5](#_ENREF_5)]. Elevation data for Namibia were extracted from Shuttle Radar Topography Mission (SRTM) data [[6](#_ENREF_6)]. The topographic wetness index, a measure representing the amount of water that should enter a given spatial unit divided by the rate at which the water should flow out of that unit, was calculated from elevation as a measure for suitability for mosquito breeding habitat [[3](#_ENREF_3),[7](#_ENREF_7),[8](#_ENREF_8)]. Moreover, gridded datasets representing distance to the nearest waterbody (Watercourse, lakes/wetlands [[9](#_ENREF_9)]) were constructed. Suitability for mosquito habitat was also described using remotely sensed imagery [[10](#_ENREF_10)]. The normalized difference vegetation index (NDVI) [[11](#_ENREF_11)] and the normalized difference water index (NDWI) [[12](#_ENREF_12)] were calculated from Moderate Resolution Imaging Spectroradiometer (MODIS) images from January-May 2011 with a spatial resolution of 250m, and 500m imagery were obtained that represented middle infrared reflectance (MIR) [[13](#_ENREF_13)]. Differing land covers provide different mosquito habitats, and therefore GlobCover land cover data [[14](#_ENREF_14)] were obtained for Namibia. Finally, population density and remoteness have been shown to be related to malaria transmission, particularly in relation to high densities in urban areas [[15](#_ENREF_15)], and therefore the AfriPop project ([www.afripop.org](http://www.afripop.org), [[16](#_ENREF_16)]) Namibia dataset was obtained and converted from population counts to densities, while road network and travel time [[17](#_ENREF_17),[18](#_ENREF_18)] datasets were used to construct distance to major road and remoteness indicators. Each of the spatial covariate datasets were resampled to 250m spatial resolution.

| **Variable** | **Input data** | **Manipulation** | **Predictors used** |
| --- | --- | --- | --- |
| Precipitation | Averages, maximums and minimums from WorldClim [[4](#_ENREF_4)] | None | Precipitation mean, minimum and maximum of December to April period. |
| Vegetation | MODIS 250m and 500m January-May 16-day composite images | Used to calculate the normalized difference vegetation index (NDVI) [[11](#_ENREF_11)] and modified normalized difference water index (NDWI) [[12](#_ENREF_12)]. | NDVI, NDWI and Middle Infrared Reflectance (MIR) mean, minimum and maximum of the January to May period |
| Waterbodies | Global Lakes and Wetlands Database (GLWD) [[9](#_ENREF_9)] | Distance to nearest watercourse, waterbody/wetland calculated. | Distance to nearest watercourse, distance to nearest waterbody/wetland. |
| Topography | Shuttle Radar Topography Mission Digital Elevation Model dataset [Ref] | Topographic Wetness Index (TWI) [[8](#_ENREF_8)] calculated from the elevation dataset. | Elevation, TWI |
| Land cover | GlobCover 300m resolution 2009 land cover dataset [[14](#_ENREF_14)] | None | Land cover |
| Temperature | Averages, maximums and minimums from WorldClim [[4](#_ENREF_4)], *P. falciparum* temperature suitability index (*Pf*TSI) [[5](#_ENREF_5)] | None | Mean, minimum and maximum of the January to May period, *Pf*TSI |
| Infrastructure | Estimated travel time to settlement of >50,000 people [[17](#_ENREF_17)], road network GIS file | Distance to nearest primary or secondary road calculated. | Remoteness, distance to major road |
| Population | Gridded population datasets from the AfriPop project [[19](#_ENREF_19)] | Conversion from population counts to densities. | Population density |

*Table 1. Spatial covariates used in the case-based risk mapping. All variables were resampled to 250m resolution.*


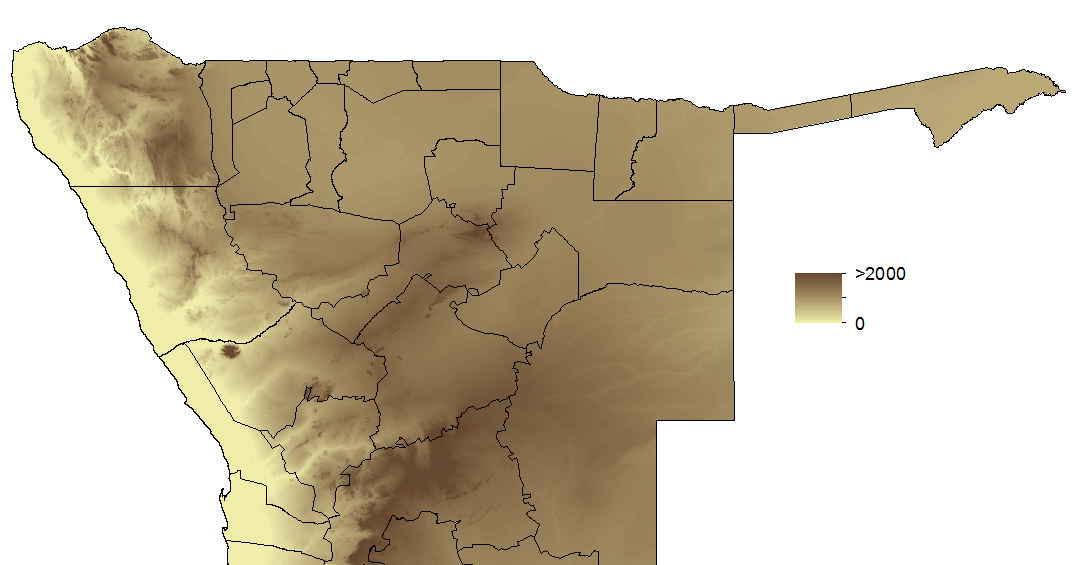

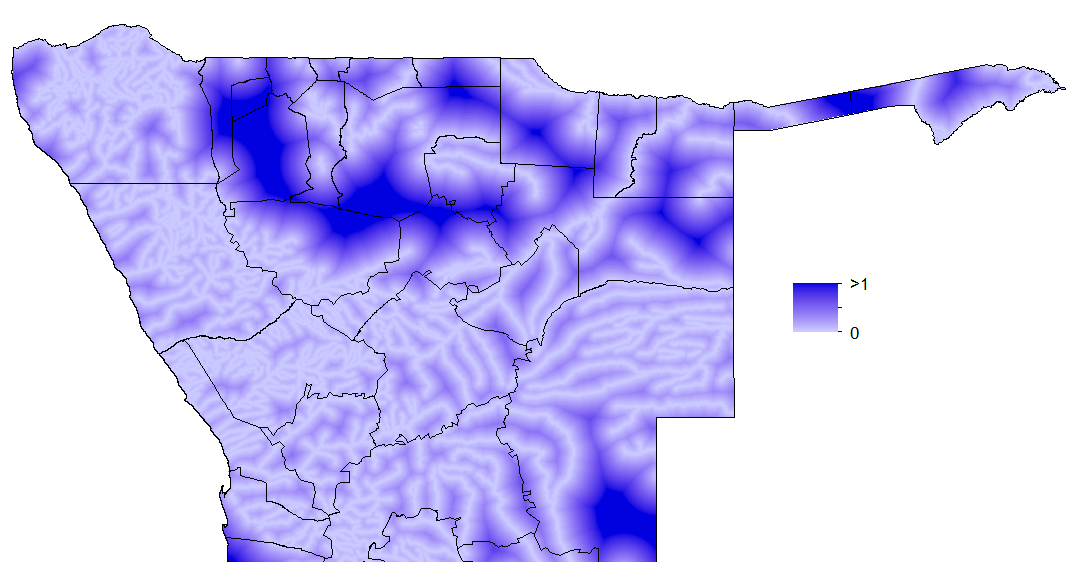
(a) (b)


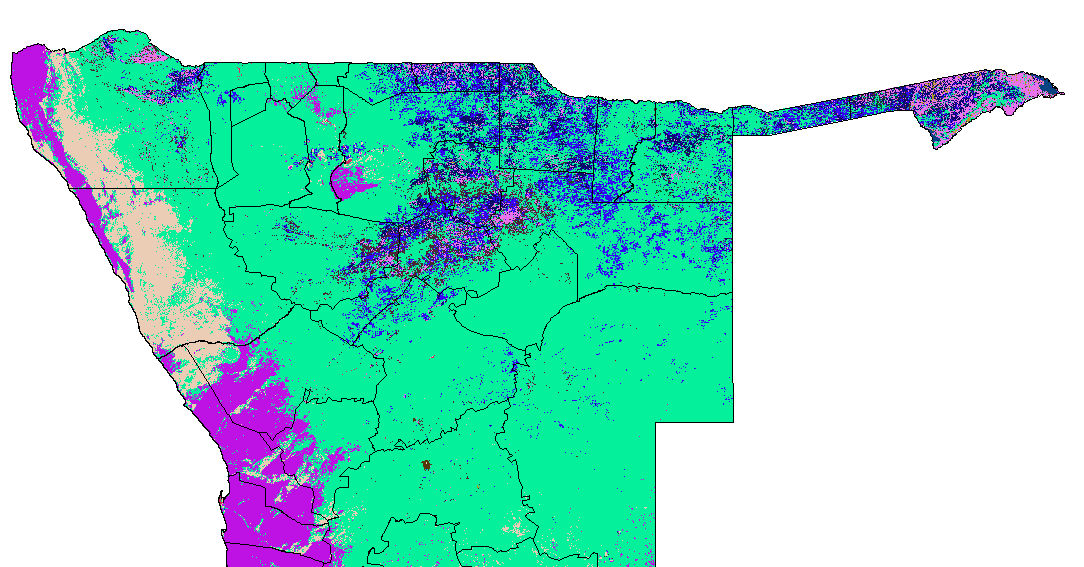

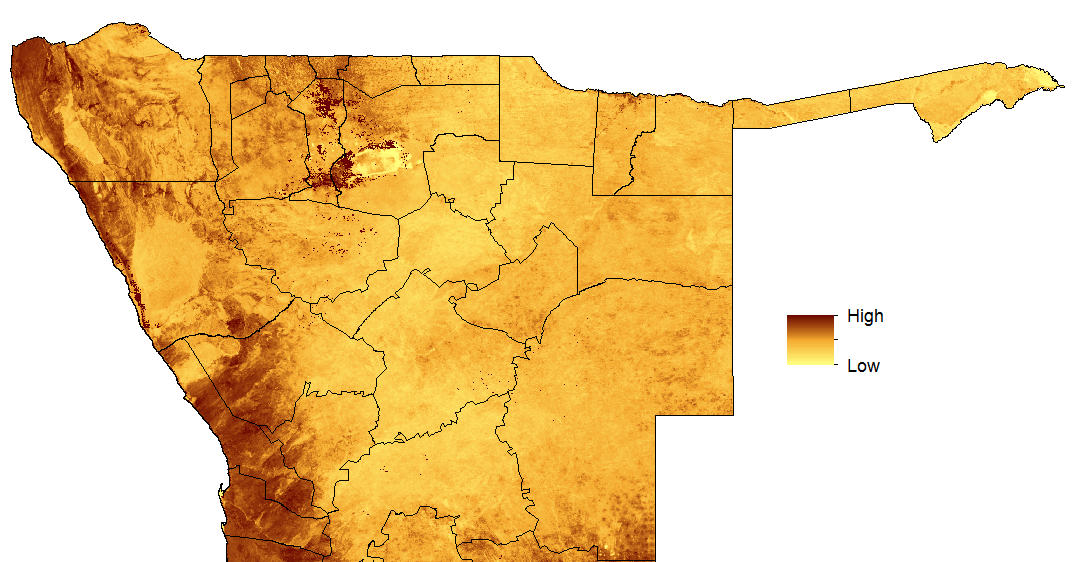


(c) (d)


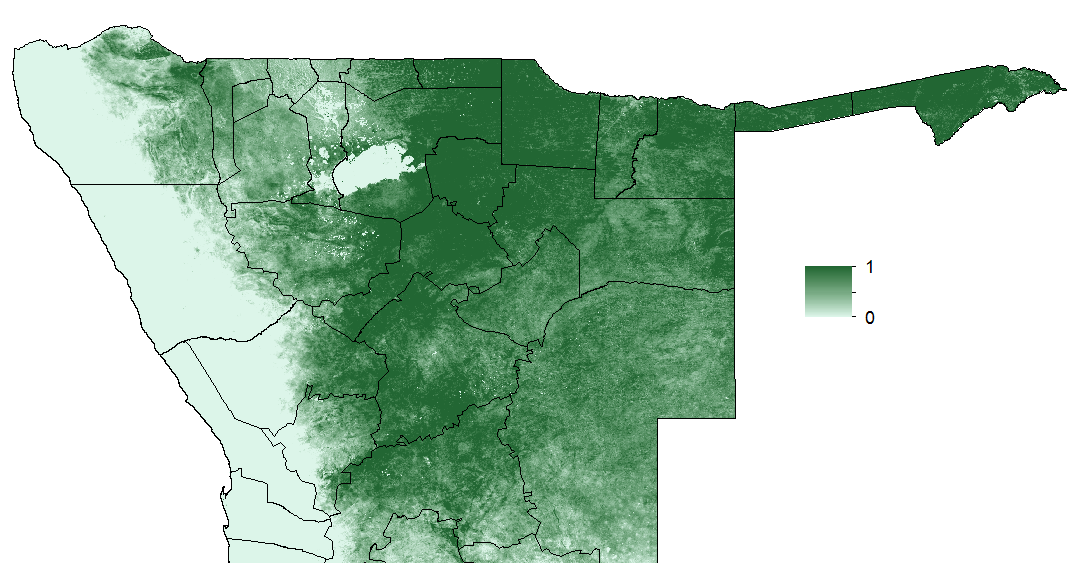

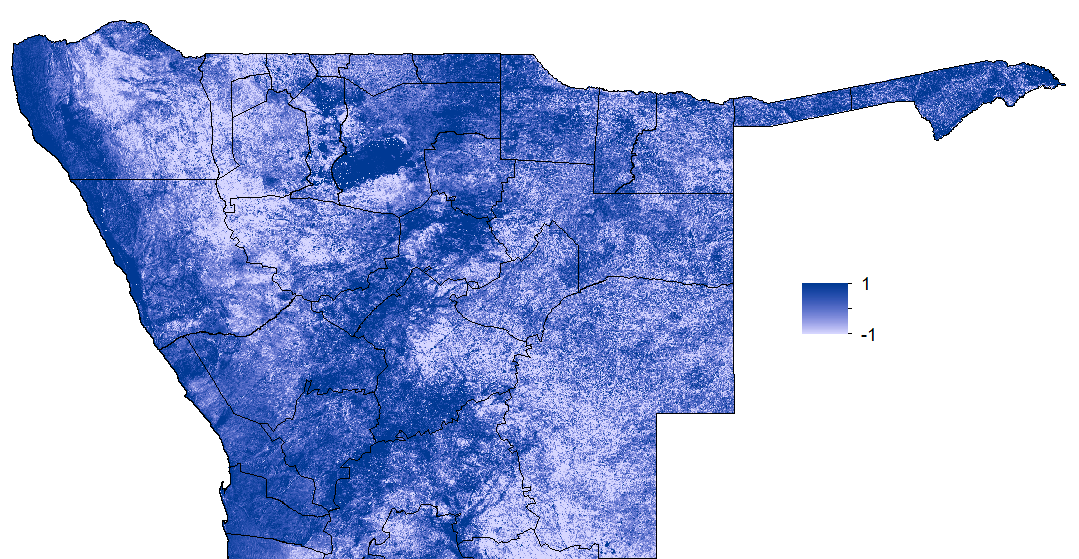


(e) (f)


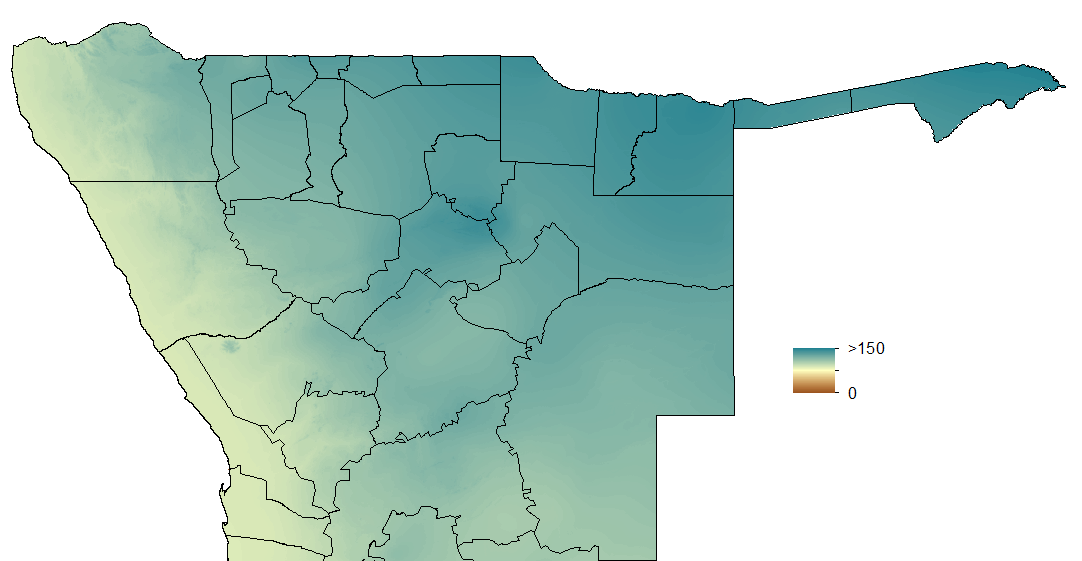

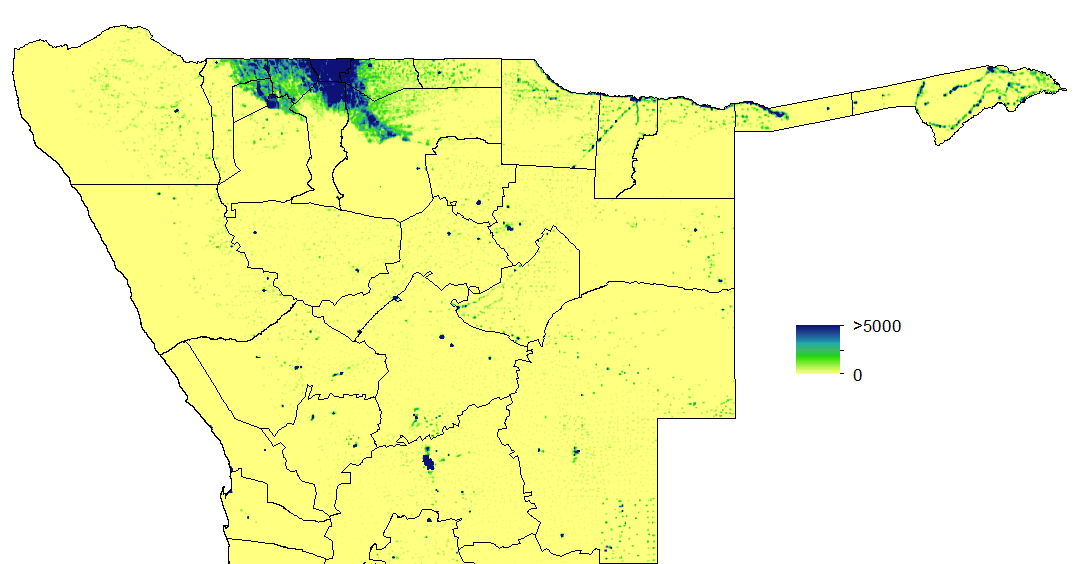


(g) (h)


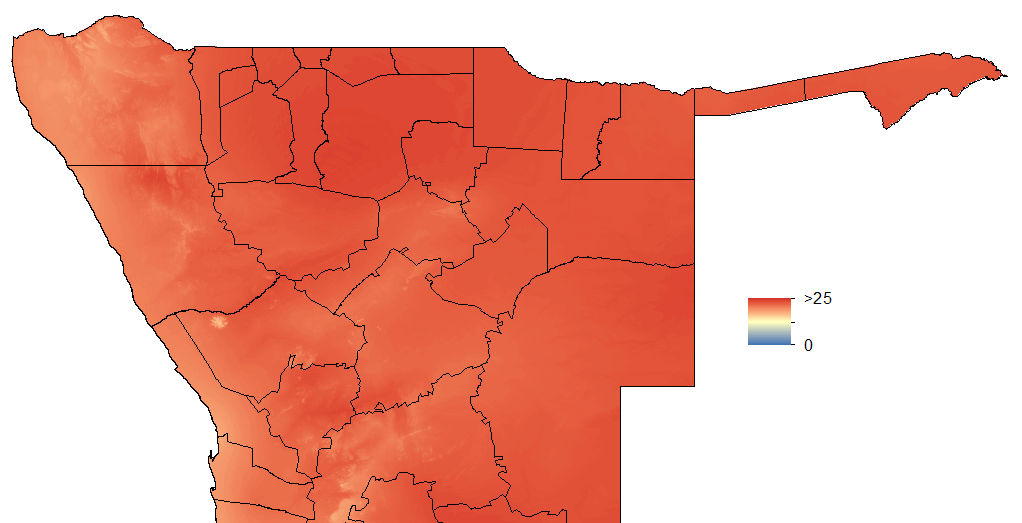

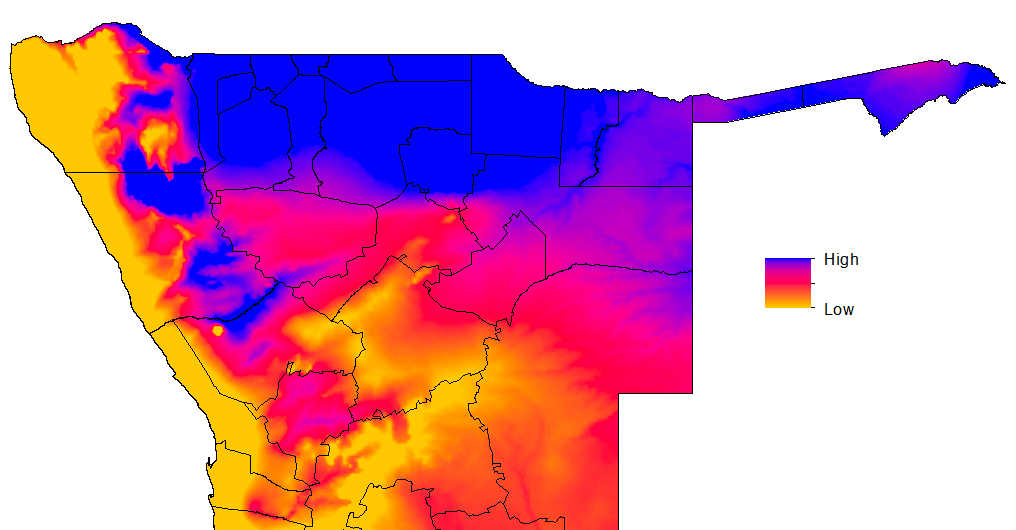


(i) (j)
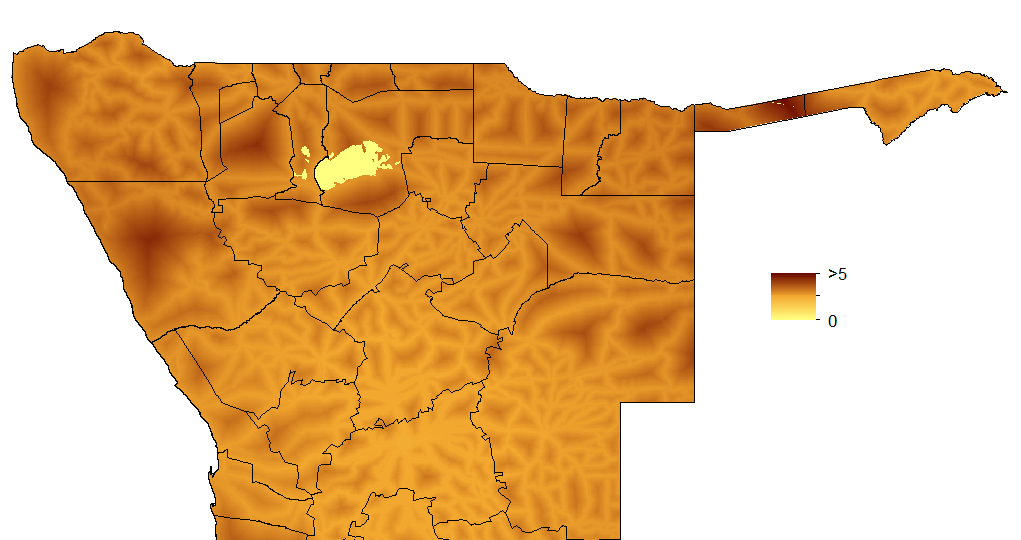

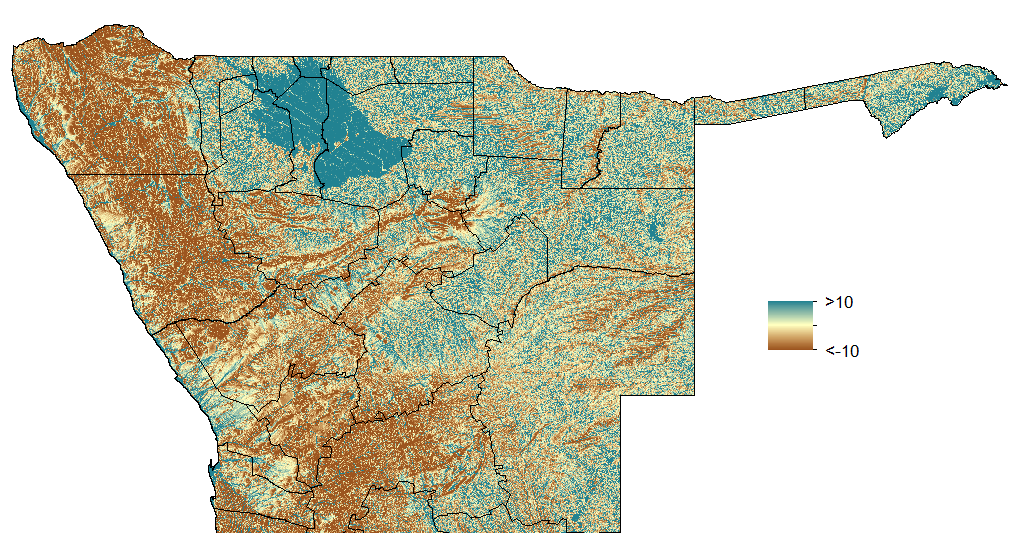


(k) (l)

Figure 1. Examples of spatial covariate datasets used: (a) Elevation; (b) Distance to the nearest watercourse; (c) Land cover; (d) Middle infrared reflectance (MIR); (e) Normalized difference vegetation index (NDVI); (f) Normalized difference water index (NDWI); (g) Mean precipitation; (h) Population density; (i) Mean temperature; (j) *P.falciparum* temperature suitability index (*Pf*TSI); (k) Travel time in hours to the nearest settlement of population > 50,000 (Remoteness); (l) Topographic wetness index (TWI). In each case, only the northern half of Namibia is shown.

***Northern Namibia case-based mapping outputs: additional details***

This section presents additional details on the northern Namibia case-based *P. falciparum* risk mapping undertaken in the main paper.

Table 2 shows the results of univariate analyses to determine the ability of each spatial covariate in distinguishing case locations from ‘background’ conditions.

| **Spatial covariate dataset** | **Cases (mean value)** | **Background (mean value)** | **t-test** | **p** |
| --- | --- | --- | --- | --- |
| Mean precipitation | 130.4 | 119.3 | -3.98 | 0.000 |
| Min precipitation | 104.3 | 74.6 | -4.11 | 0.000 |
| Max precipitation | 146.2 | 131.7 | -3.49 | 0.000 |
| Mean temperature | 25.6 | 25.5 | -0.72 | 0.421 |
| Min temperature | 19.3 | 19.1 | -0.91 | 0.327 |
| Max temperature | 28.0 | 27.9 | -0.84 | 0.501 |
| Elevation | 1141 | 1176 | 1.12 | 0.402 |
| TWI | -8.1 | -4.9 | 2.44 | 0.008 |
| Mean NDVI | 0.61 | 0.54 | -4.01 | 0.000 |
| Min NDVI | 0.52 | 0.45 | -4.25 | 0.000 |
| Max NDVI | 0.69 | 0.62 | -3.77 | 0.000 |
| Mean NDWI | -0.16 | -0.05 | 2.17 | 0.000 |
| Min NDWI | -2.04 | -1.67 | 1.98 | 0.000 |
| Max NDWI | 0.91 | 0.44 | 2.50 | 0.000 |
| Mean MIR | 28.3 | 18.1 | -6.01 | 0.000 |
| Min MIR | 21.2 | 16.5 | -5.66 | 0.000 |
| Max MIR | 33.7 | 29.9 | -4.06 | 0.000 |
| Distance to lake/wetland | 0.03 | 0.05 | 2.81 | 0.001 |
| Distance to watercourse | 0.02 | 0.04 | 3.83 | 0.000 |
| Population density | 33.4 | 12.5 | -6.76 | 0.000 |
| Remoteness | 503 | 655 | 0.42 | 0.489 |
| Dist to nearest major road | 0.02 | 0.03 | 0.27 | 0.647 |
| *Pf*TSI | 36322 | 36408 | 0.98 | 0.102 |

Table 2. Characteristics of the locations of settlements with cases compared against the characteristics of 10,000 population-weighted random background locations. Full details on the spatial covariates used are provided in the previous section.

All of the predictor variables were included in the fitting step to produce a model predicting the probability of cases occurring at a particular location as a function of the combined covariates. Model quality was assessed by examining calibration plots [[20](#_ENREF_20)], the area under the curve (AUC) on receiver operating characteristic (ROC) graphs and correlation statistics [[21](#_ENREF_21)]. The area under the curve (AUC) of the receiver-operator characteristic (ROC) is also presented as an accuracy metric. The AUC provides values ranging between 0 and 1, where 1 is a perfect prediction, and is an index of the area under the curve of a plot of sensitivity (the proportion that is correctly classified as a presence) against specificity (the proportion that is correctly classified as an absence) as the classification threshold is increased [[21](#_ENREF_21)]. Correlation simply correlates the predicted probability of presence against the case occurrence data, giving a value between -1 and 1.

Figure 2 shows the (a) ROC and (b) calibration plots for the final model. The ROC plot suggests strong model prediction, with AUC = 0.96, while the calibration plot suggests that the model was not biased as observed standard errors overlap the diagonal (Figure 2b). The correlation between the predicted probabilities of presence against the case occurrence data was 0.82.


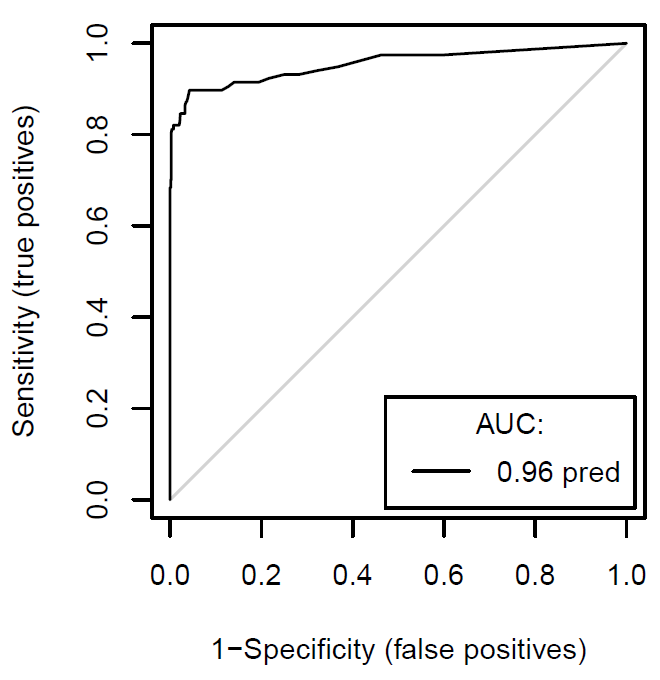

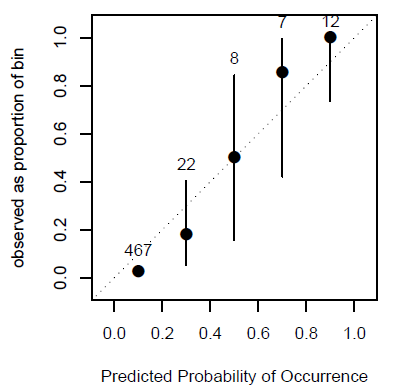


(a) (b)

*Figure 2. (a) Receiver-operator characteristic (ROC) and (b) calibration plots for the northern Namibia case-based risk mapping shown in the main paper.*

The relative influence of each covariate dataset in building the northern Namibia model are shown in table 3, highlighting that stronger dependence upon NDVI, population density, distance to watercourse and NDWI was observed than for temperature, elevation, distance to nearest major road and remoteness.

| **Spatial covariate dataset** | **Relative influence (%)** |
| --- | --- |
| Mean precipitation | 5.0 |
| Min precipitation | 5.1 |
| Max precipitation | 4.3 |
| Mean temperature | 0.2 |
| Min temperature | 0.2 |
| Max temperature | 0.1 |
| Elevation | 0.1 |
| TWI | 3.4 |
| Mean NDVI | 8.7 |
| Min NDVI | 9.2 |
| Max NDVI | 7.1 |
| Mean NDWI | 5.4 |
| Min NDWI | 5.1 |
| Max NDWI | 5.2 |
| Mean MIR | 8.9 |
| Min MIR | 7.3 |
| Max MIR | 6.2 |
| Distance to lake/wetland | 3.0 |
| Distance to watercourse | 4.3 |
| Population density | 9.0 |
| Remoteness | 0.4 |
| Dist to nearest major road | 0.3 |
| *Pf*TSI | 1.0 |
| Land Cover | 0.4 |

*Table 3. Relative influence of spatial covariates, shown as percentages.*

***Individual district mapping and cross-comparisons***

The construction of a case-based risk map for the entire northern Namibia region from confirmed case data from three districts introduces uncertainty into the mapping process through spatial extrapolation to other districts. Moreover, the possibility remains that different factors drive the appearance of cases between districts. Therefore, to examine (i) the accuracy of mapping through spatial extrapolation and (ii) the consistency in driving factors, random forest models were built for each individual district using the same set of spatial covariates as described earlier and tested on their abilities to accurately identify case locations in the other districts for which case data were available. For each district model 5000 background points were used. Model fits for the individual districts were, unsurprisingly, poorer than the model built using all available case data across the three districts, as presented in the main paper, and this was most likely due to the smaller sample sizes when examining individual districts. Nevertheless, the statistics still indicate relatively strong model prediction abilities, with consistently high values across the three districts (table 4).

| **District** | **AUC** | **Correlation** |
| --- | --- | --- |
| Caprivi | 0.88 | 0.69 |
| Kavango | 0.91 | 0.71 |
| Omusati | 0.79 | 0.60 |

*Table 4. AUC and correlation statistics for the models constructed for (a) Caprivi, (b) Kavango and (c) Omusati.*

Table 5 shows the spatial covariate contributions, ranked for ease of comparison, and highlights the relative consistency in apparent driving factors in determining case locations between the three districts. While differences were apparent, particularly between Omusati and the other two districts, for all three districts stronger dependence upon NDVI, population density, distance to watercourse, MIR, precipitation and NDWI were seen, but weaker dependence on temperature, elevation, distance to nearest major road and remoteness, mirroring results from the combined district model.

| **Spatial covariate dataset** | **Caprivi** | **Kavango** | **Omusati** |
| --- | --- | --- | --- |
| Mean precipitation | 12 | 14 | 11 |
| Min precipitation | 7 | 7 | 10 |
| Max precipitation | 14 | 12 | 14 |
| Mean temperature | 21 | 22 | 21 |
| Min temperature | 20 | 21 | 20 |
| Max temperature | 23 | 24 | 22 |
| Elevation | 22 | 23 | 24 |
| TWI | 13 | 15 | 7 |
| Mean NDVI | 3 | 3 | 1 |
| Min NDVI | 1 | 1 | 2 |
| Max NDVI | 5 | 4 | 5 |
| Mean NDWI | 9 | 8 | 9 |
| Min NDWI | 8 | 9 | 12 |
| Max NDWI | 10 | 10 | 13 |
| Mean MIR | 4 | 6 | 3 |
| Min MIR | 6 | 5 | 4 |
| Max MIR | 11 | 11 | 8 |
| Distance to lake/wetland | 16 | 17 | 23 |
| Distance to watercourse | 15 | 13 | 17 |
| Population density | 2 | 2 | 6 |
| Remoteness | 18 | 18 | 15 |
| Dist to nearest major road | 19 | 19 | 16 |
| *Pf*TSI | 17 | 16 | 18 |
| Land Cover | 24 | 20 | 19 |

*Table 5.* *Ranking of each spatial covariate in terms of relative contribution to model (1=largest contribution) for each district model.*

Table 6 shows the results of district mapping cross-comparisons, where a RF model was constructed based on the case data from two districts and used to predict case locations in the other one. The results show generally good predictive capacity, reflecting the relative consistency in driving factors across northern Namibia. For Omusati, predictions were weaker, likely reflecting some of the differences in driving factors seen in table 5 compared to the other two districts.

| **Model training districts** | **Model testing district** | **AUC** | **Correlation** |
| --- | --- | --- | --- |
| Omusati and Kavango | Caprivi | 0.84 | 0.66 |
| Omusati and Caprivi | Kavango | 0.86 | 0.67 |
| Kavango and Caprivi | Omusati | 0.71 | 0.51 |

*Table 6. AUC and correlation statistics for models built on case data from two districts and used to predict in the third district.*

***Mapping undertaken without isolated cases***

As travel history information from confirmed cases was not collected, tests were undertaken to attempt to control for the fact that patients may have obtained infections away from their place of residence, locations with (i) just one case, then (ii) with one or two cases, were dropped, based on the assumption that multiple cases in a location are more likely to be representative of local transmission, and the output results compared to the mapping run using all case data to examine how sensitive outputs were to the exclusion of these isolated cases.

Removing locations where just one case was reported reduced the number of unique locations considered from 162 to 140, and removing locations where either one or two cases were reported reduced the number to 129. Model fits were marginally poorer for both sets of data compared to the model presented in the main paper using all data, and this was likely due at least in part to having fewer training locations. The results of the ROC plots and output AUCs are presented in figure 4 below. The AUC and correlation statistics were 0.95 and 0.79 when removing locations where just one case was reported and 0.94 and 0.77 when removing locations with one or two cases. Output maps showed no clear visual differences from those shown in figure 1 of the main paper.


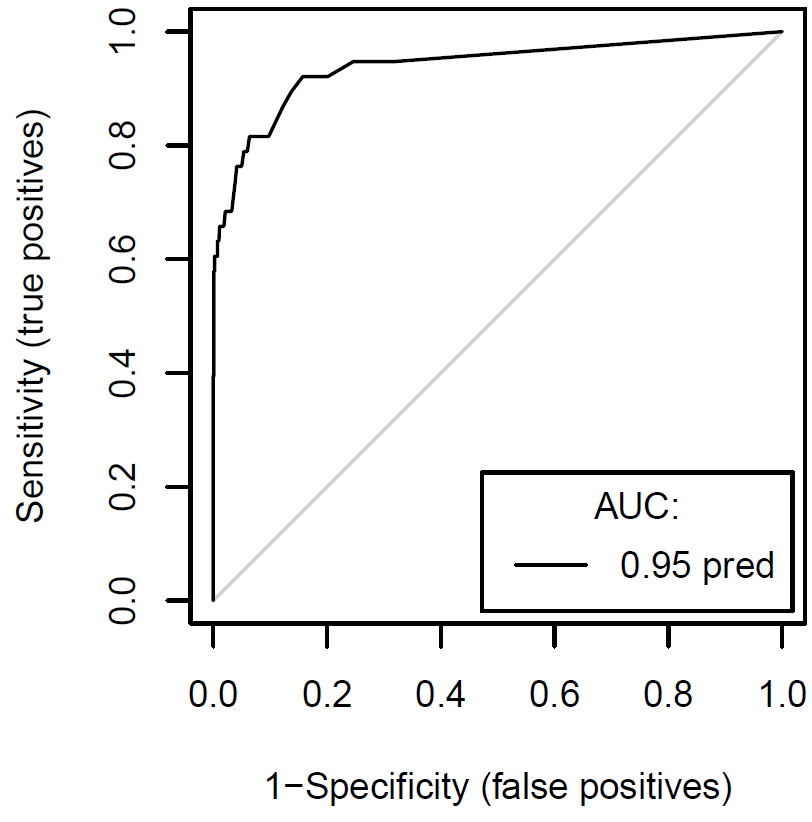

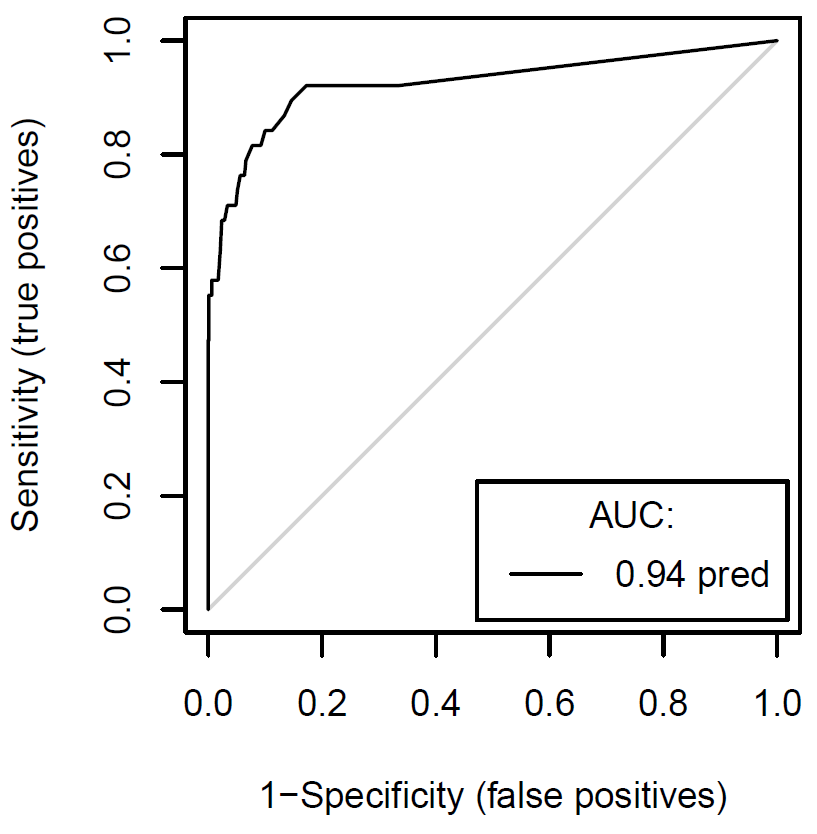


1. (b)

*Figure 4. Receiver-operator characteristic (ROC) plots for the models constructed through removing locations with (a) just one case and (b) one or two cases.*

***References***

1. Cohen JM, Dlamini SS, Novotny JM, Kandula D, Kunene S, et al. (2013) Rapid case-based mapping of seasonal malaria transmission risk for strategic elimination planning in Swaziland. Malar Journal 12: 61.

2. Craig MH, Snow RW, le Sueur D (1999) A climate-based distribution model of malaria transmission in sub-Saharan Africa. Parasitol Today 15: 105-111.

3. Cohen JM, Ernst KC, Lindblade KA, Vulule JM, John CC, et al. (2008) Topography-derived wetness indices are associated with household-level malaria risk in two communities in the western Kenyan highlands. Malar J 7: 40.

4. Hijmans RJ, Cameron SE, Parra JL, Jones PG, Jarvis A (2005) Very high resolution interpolated climate surfaces for global land areas International Journal of Climatology 25: 1965-1978.

5. Gething PW, Van Boeckel TP, Smith DL, Guerra CA, Patil AP, et al. (2011) Modelling the global constraints of temperature on transmission of Plasmodium falciparum and P. vivax. Parasit Vectors 4: 92.

6. Farr TG, Rosen PA, Caro E, Crippen R, Duren R, et al. (2007) The Shuttle Radar Topography Mission. Reviews of Geophysics 45.

7. Cohen JM, Ernst KC, Lindblade KA, Vulule JM, John CC, et al. (2010) Local topographic wetness indices predict household malaria risk better than land-use and land-cover in the western Kenya highlands. Malar J 9: 328.

8. Sorensen R, Zinko U, Seibert J (2006) On the claculation of the topographic wtness index: evaluation of idfferent methods based on field observations. Hydrology and Earth System Sciences 10: 101-112.

9. Lehner B, Doll P (2004) Development and validation of a global database of lakes, reservoirs and wetlands. Journal of Hydrology 296: 1-22.

10. Hay SI, Snow RW, Rogers DJ (1998) From predicting mosquito habitat to malaria seasons using remotely sensed data: practice, problems and perspectives. Parasitol Today 14: 306-313.

11. Tucker CJ (1979) Red and photographic infrared linear combinations for monitoring vegetation. Remote Sensing of Environment 8: 127-150.

12. Xu H (2006) Modification of normalized difference water index (NDWI) to enhance open water features in remotely sensed imagery. International Journal of Remote Sensing 27: 3025-3033.

13. Boyd DS, Curran PJ (1998) Using remote sensing to reduce uncertainties in the global carbon budget: the potential of radiation acquired in middle infrared wavelengths. Remote Sensing Reviews 16: 293-327.

14. Arino O, Bicheron P, Achard F, Latham J, Witt R, et al. (2008) GLOBCOVER: The most detailed portrait of Earth. European Space Agency 136: 24-31.

15. Tatem AJ, Guerra CA, Kabaria CW, Noor AM, Hay SI (2008) Human population, urban settlement patterns and their impact on *Plasmodium falciparum* malaria endemicity. Malaria Journal 7: 218.

16. Linard C, Gilbert M, Snow RW, Noor AM, Tatem AJ (2012) Population Distribution, Settlement Patterns and Accessibility across Africa in 2010. PLoS ONE 7.

17. Nelson A (2008) Estimated travel time to the nearest city of 50,000 or more people in year 2000. Ispra, Italy: Global Environment Monitoring Unit, Joint Research Centre.

18. Tatem AJ, Hemelaar J, Gray RR, Salemi M (2012) Spatial accessibility and the spread of HIV-1 subtypes and recombinants. Aids 26: 2351-2360.

19. Linard C, Gilbert M, Snow RW, Noor AM, Tatem AJ (2012) Population distribution, settlement patterns and accessibility across Africa in 2010. PLoS ONE 7: e31743.

20. Pearce F, Ferrier S (2000) Evaluating the predictive performance of habitat models developed using logistic regression. Ecological Modelling 133: 225-245.

21. Rogers DJ (2006) Models for vectors and vector-borne diseases. Adv Parasitol 62: 1-35.
