# Supplementary material for: Integrating rapid risk mapping and mobile phone call record data for strategic malaria elimination planning
Source: Malar J. 2014 Feb 10;13:52. doi: 10.1186/1475-2875-13-52 (PMC3927223; doi:10.1186/1475-2875-13-52)
Supplement: Additional file 2 — Mobile phone call detail records – additional details. Further information on phone ownership, mobile phone network geography and mobility patterns in Namibia. [file 1475-2875-13-52-S2.docx]

**Additional File 2: Mobile phone call detail records – additional details**

***The demographics of mobile phone ownership in Namibia***

In 2011, 56.2% of the population of Namibia were mobile phone owners, with the percentage ownership in urban areas being 76.7% and 46.1% in rural areas [[1](#_ENREF_1)]. To ascertain demographic features of phone ownership in Namibia, data from a national survey of over 22,000 people undertaken in 2011 were analysed [[1](#_ENREF_1)]. Figure 1 shows the age distribution of those surveyed (over 15 years old only) and their mobile phone ownership status, highlighting that the percentage of those owning phones remains relatively constant by age.


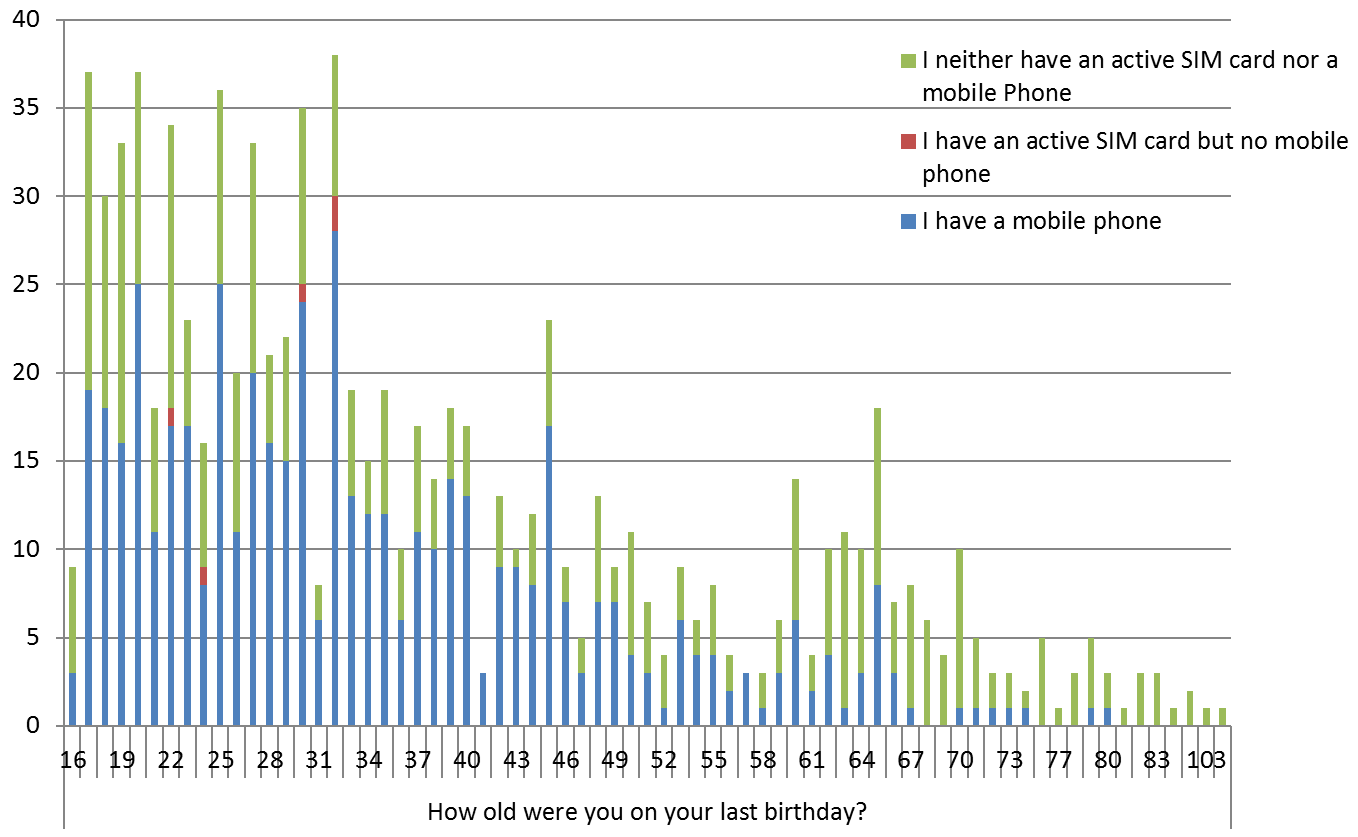


*Figure 1. Phone and SIM card ownership by age for those questioned in a recent national survey [*[*1*](#_ENREF_1)*].*

Figure 2 shows phone ownership status by activity in the 6 months prior to survey, and highlights the substantially higher ownership rates in those who were employed versus those who were unemployed or retired. This indicates that the call detail records (CDRs) analysed in this study are, unsurprisingly, likely to be representing a partially biased subset of the population, probably towards those who are wealthier and more mobile, reflecting a similar bias in phone ownership seen in an earlier survey [[2](#_ENREF_2)], towards wealthier people being more likely to be phone owners.


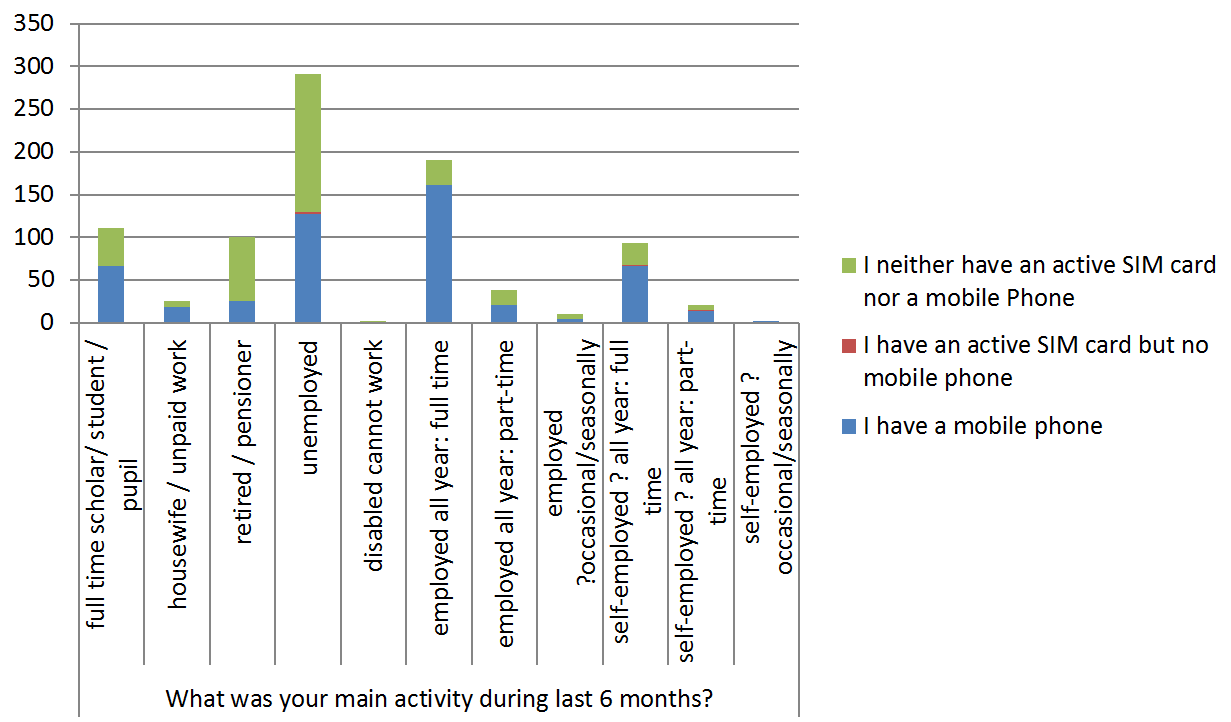


*Figure 2. The main activity of those surveyed [*[*1*](#_ENREF_1)*] and their phone / Sim card ownership status.*

Finally, figure 3 shows that phones are often shared between friends and family members, highlighting that the movements captured in the analyses presented may sometimes not be representative of the same individual.


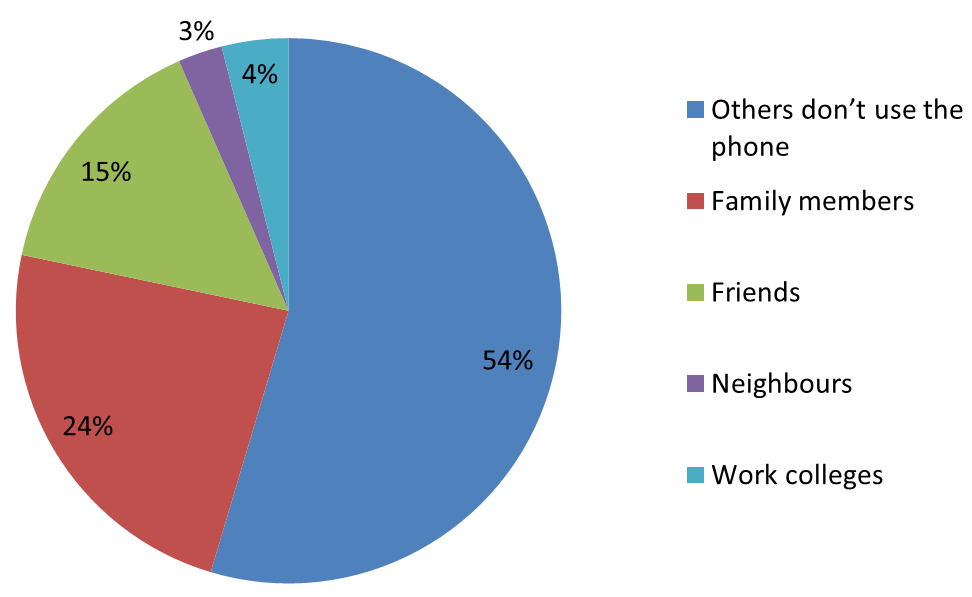


*Figure 3. Phone sharing characteristics of those questioned in a recent survey* [[1](#_ENREF_1)].

***Mobile phone network geography***


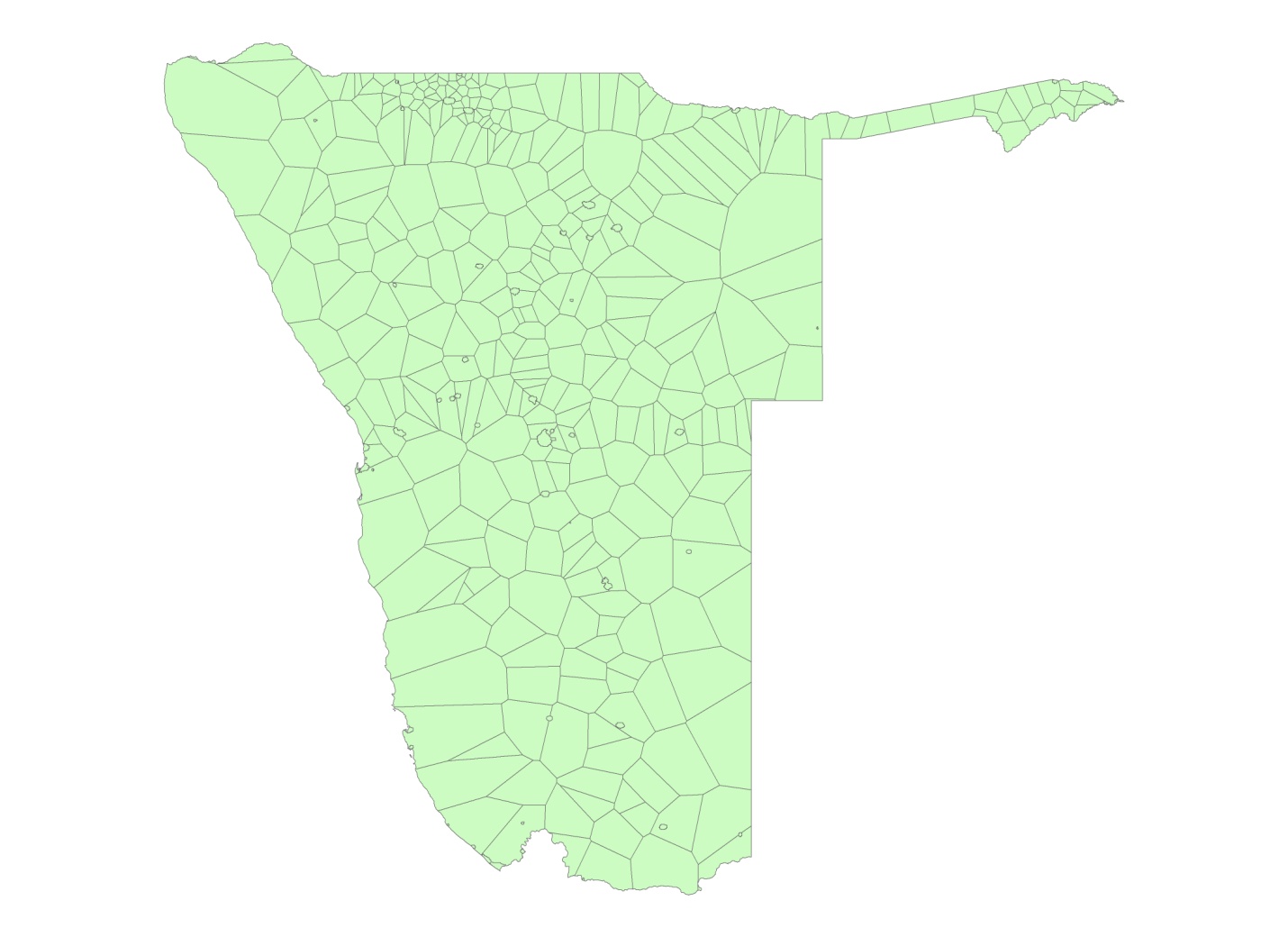


*Figure 4. Phone coverage areas used in the analyses presented, calculated as Voronoi tesselations around towers, with urban polygons defined using a separate urban extent layer [*[*3*](#_ENREF_3)*].*

Each call/text made in the CDRs was routed through the nearest phone tower to the user. The distribution of towers (that strongly follows population density [[4](#_ENREF_4)]) thus determined the precision with which a user’s location could be determined. Following previous studies [[4](#_ENREF_4),[5](#_ENREF_5)], Voronoi tesselations were used to convert tower locations to phone coverage areas to provide areal representations of the possible locations of each user, with towers defined as being in urban areas if they fell within a pre-defined urban extent [[3](#_ENREF_3)] and matched to these extents (Figure 4). These areas were then further refined by broad masking of regions where coverage was reported to be unavailable: <http://www.mtc.com.na/coverage>***.***

***Population mobility patterns***


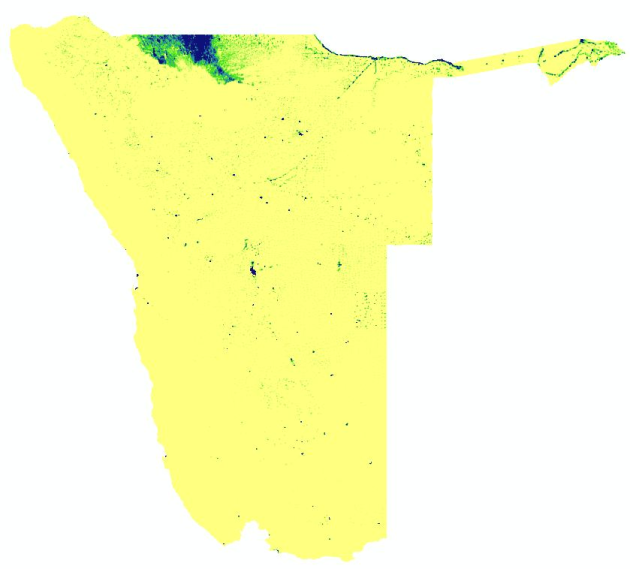

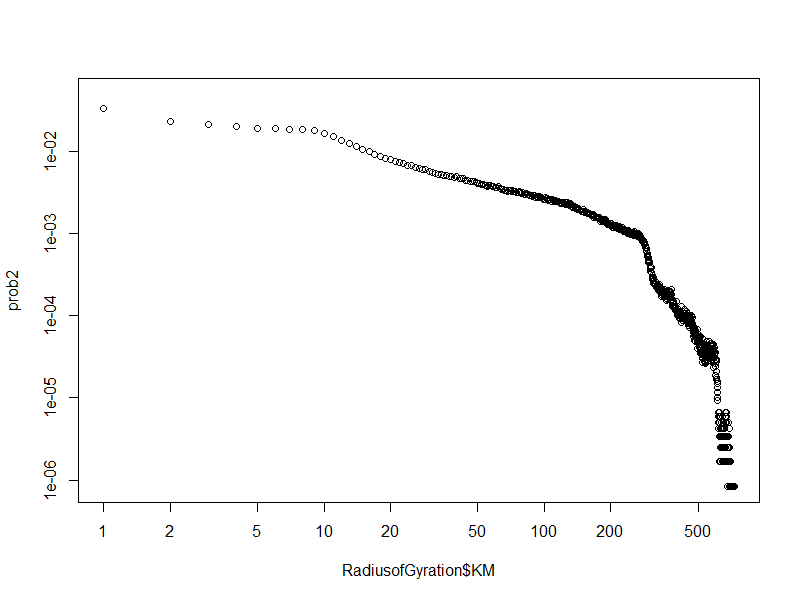


(a)


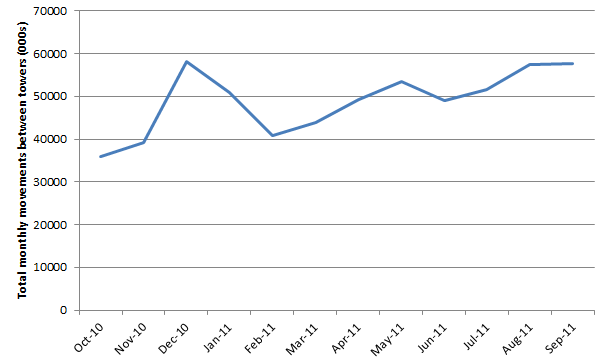


(b)

*Figure 5. (a) Plot of radius of gyration for all movements in Namibia, with a population distribution map inset; (b) The numbers of movements between phone towers measured per month for the Oct 2010-Sept 2011 period.*

Analyses of radius of gyration show that population movements in Namibia follow patterns seen elsewhere [[5-7](#_ENREF_5)] of shorter distance movements being substantially more common than larger ones and more isolated populations generally travelling further than those in densely populated areas (Figure 5a). However, while many prior studies have found movement patterns from CDRs following smooth power-law distributions, the population distribution of Namibia results in a deviation from this pattern at a distance of around 400km, corresponding to the distance apart of the two major regions of high population density, Windhoek and the northern region (Figure 5a, inset). Across the 12-month period examined, a broad trend of increasing movement is evident (Figure 5b), corresponding to increasing numbers of SIM cards, with some seasonality in overall movement rates evident, including increased activity in December, just before the main malaria transmission season, as well as peaks around May and August.


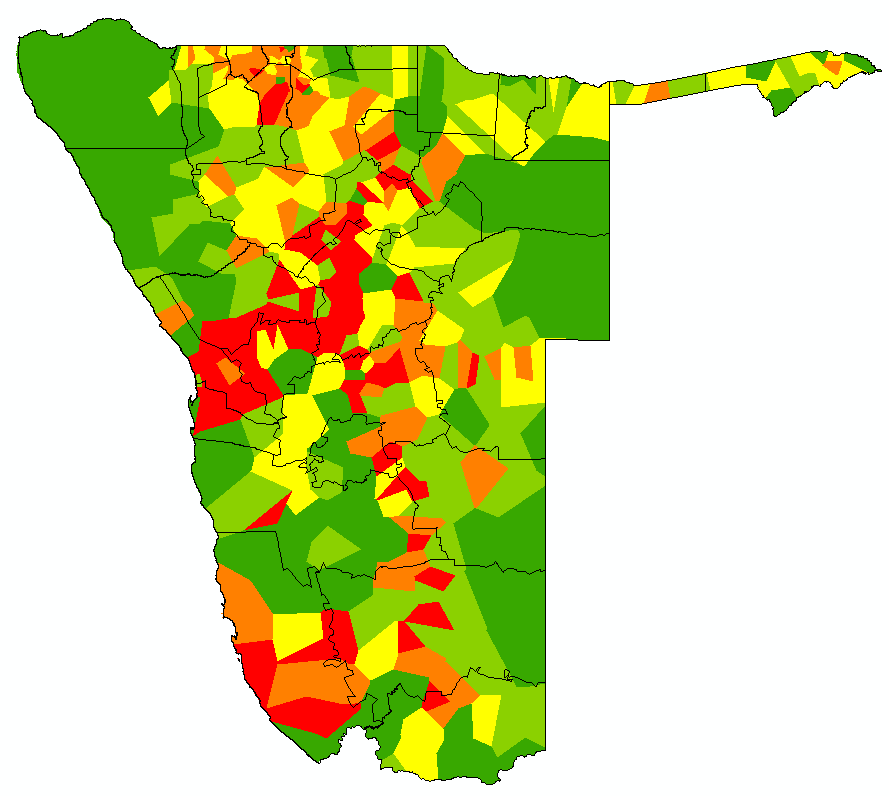

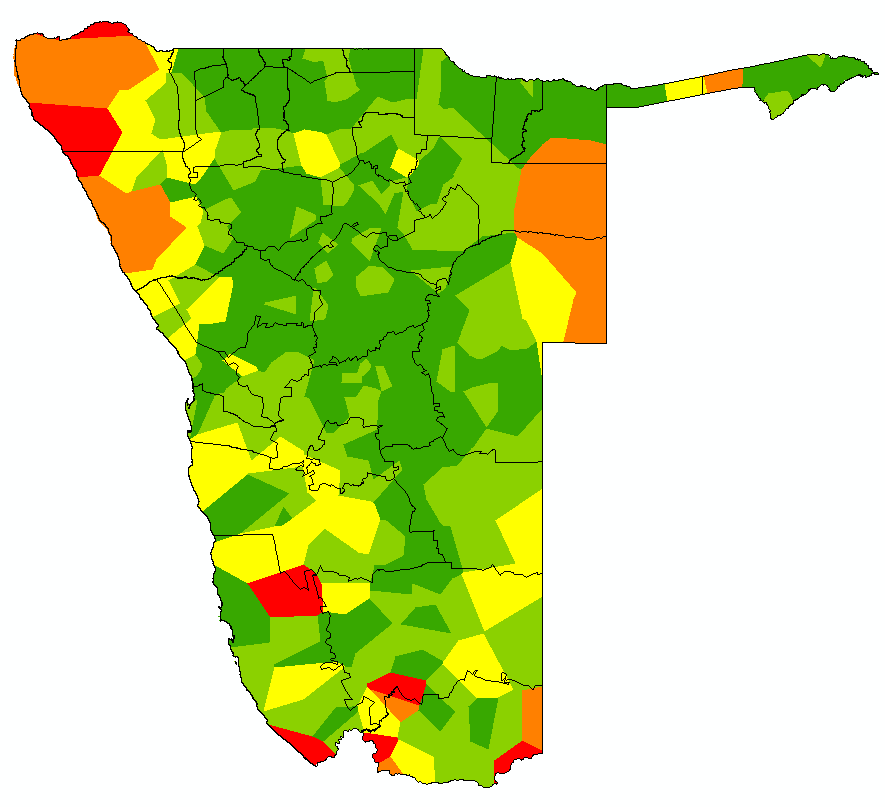


1. (b)

*Figure 6.(a) total trips made to other phone catchment areas per capita residential population; (b) mean length of trip per phone catchment. In each map the outline of health districts are overlaid. In both cases, red signifies the highest values and green represents the lowest values, with intermediate values in yellow.*

Figure 6 shows the contrasting features of population mobility between the urban, higher population density and more connected regions of the country, and the rural, low density, inaccessible areas. Those higher population density areas that are better connected through the major road networks make substantially more trips per-capita than the more rural and inaccessible areas (Figure 6a). However, unsurprisingly, when trips are made from the more remote areas, they tend to be longer in terms of length of time away than for the more populated regions (Figure 6b).

*
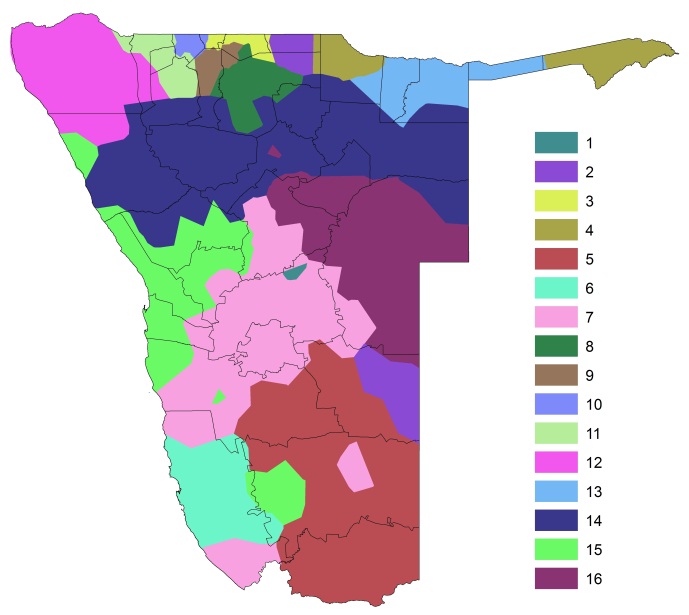

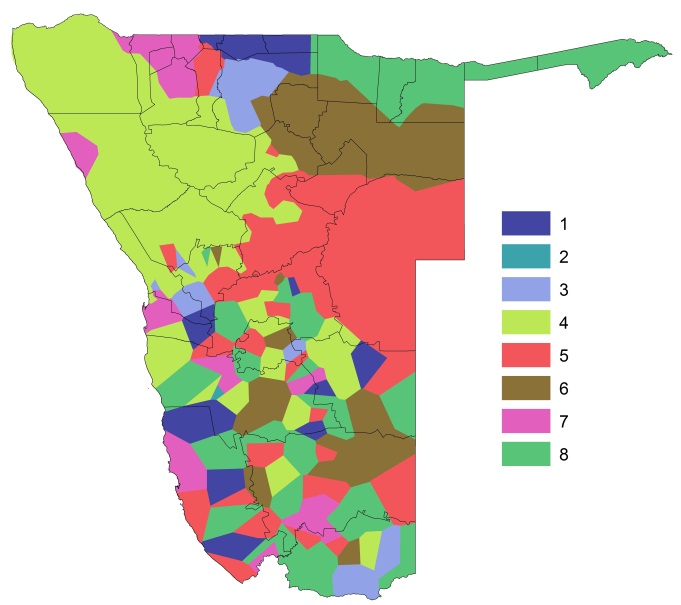
*

1. (b)

*Figure 7. Mapped communities for (a) human travel and (b) malaria case risk connectivity. In (a) and (b), regions mapped with the same colour are those for which connectivity within them are stronger than connectivity between surrounding communities .The community numbers match those in table 1 in the main paper.*

Figure 7 shows those areas estimated to encompass significant interchange of people and infections. Community detection applied to the weighted network of movements between the 402 phone catchment areas resulted in 16 communities of areas being found, and these are mapped in figure 7a, with each community shown in a single colour. The pattern of communities for human movement is very different to that identified for predicted relative infection movements (figure 7b). Unsurprisingly, little spatially coherent structure is evident in the south of the country where there is no malaria transmission and therefore zero relative infection risk movements between areas predicted.


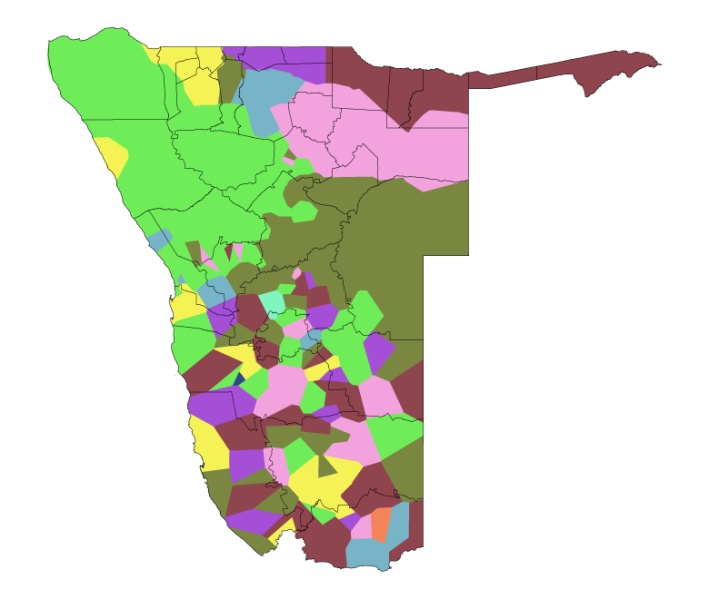

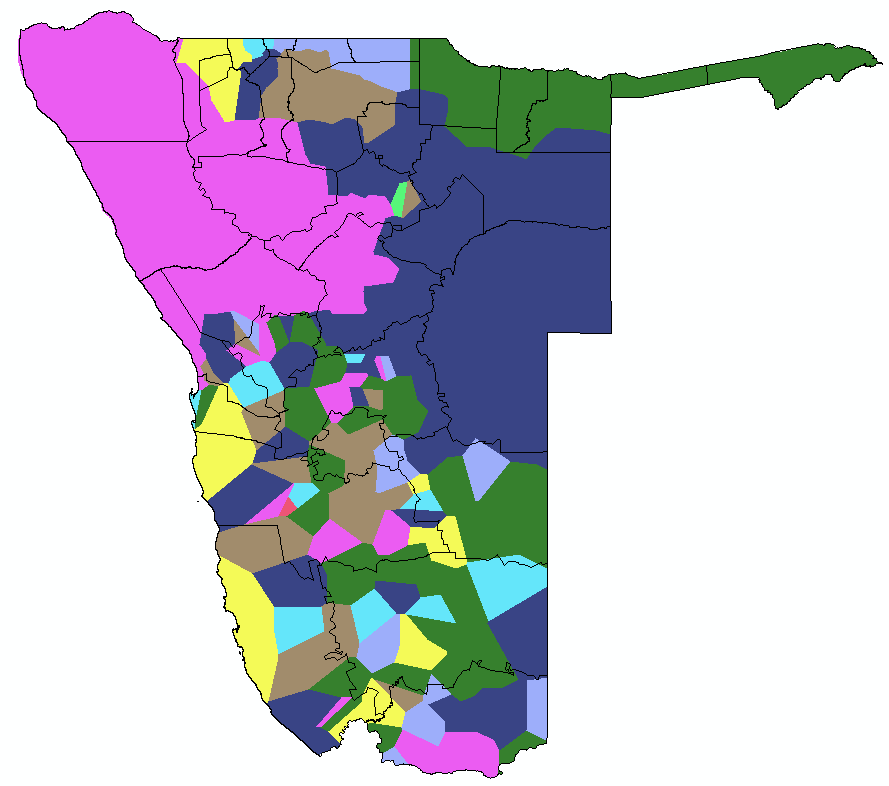


1. (b)

*Figure 8. Malaria risk connectivity network community detection for (a) returning residents, and (b) visitors. In each map the outline of health districts are overlaid.*

Finally, figure 8 shows the differences in malaria risk connectivity network community detection between returning residents and visitors, providing a breakdown of the overall communities presented in the main document.

**References**

1. Communications Regulatory Authority of Namibia (2011) Universal Service Baseline Study. Windhoek: Communications Regulatory Authority of Namibia,.

2. MEASURE DHS (2006) Namibia Demographic and Health Survey. Washington DC: USAID.

3. Balk DL, Deichmann U, Yetman G, Pozzi F, Hay SI, et al. (2006) Determining global population distribution: methods, applications and data. Advances in Parasitology 62: 119-156.

4. Wesolowski A, Eagle N, Tatem AJ, Smith DL, Noor AM, et al. (2012) Quantifying the impact of human mobility on malaria. Science 338: 267-270.

5. Gonzalez MC, Hidalgo CA, Barabasi AL (2008) Understanding individual human mobility patterns. Nature 453: 779-782.

6. Simini F, Gonzalez MC, Maritan A, Barabasi AL (2012) A universal model for mobility and migration patterns. Nature 484: 96-100.

7. Song C, Qu Z, Blumm N, Barabasi AL (2010) Limits of predictability in human mobility. Science 327: 1018-1021.
